# Allelic heterogeneity of *TTNtv* dilated cardiomyopathy can be modeled in adult zebrafish

**DOI:** 10.1172/jci.insight.175501

**Published:** 2024-02-27

**Authors:** Ping Zhu, Jiarong Li, Feixiang Yan, Shahidul Islam, Xueying Lin, Xiaolei Xu

**Affiliations:** 1 Department of Biochemistry and Molecular Biology and; 2 Department of Cardiovascular Medicine, Mayo Clinic, Rochester, Minnesota, USA.; 3 Department of Cardiovascular Surgery, Second Xiangya Hospital, Central South University, Changsha, China.

**Keywords:** Cardiology, Genetics, Autophagy, Cardiovascular disease, Genetic diseases

## Abstract

Allelic heterogeneity (AH) has been noted in truncational *TTN*–associated (*TTNtv*-associated) dilated cardiomyopathy (DCM); i.e., mutations affecting A-band–encoding exons are pathogenic, but those affecting Z-disc–encoding exons are likely benign. The lack of an in vivo animal model that recapitulates AH hinders the deciphering of the underlying mechanism. Here, we explored zebrafish as a candidate vertebrate model by phenotyping a collection of zebrafish *ttntv* alleles. We noted that cardiac function and sarcomere structure were more severely disrupted in *ttntv-A* than in *ttntv-Z* homozygous embryos. Consistently, cardiomyopathy-like phenotypes were present in *ttntv-A* but not *ttntv-Z* adult heterozygous mutants. The phenotypes observed in *ttntv-A* alleles were recapitulated in null mutants with the full *titin*-encoding sequences removed. Defective autophagic flux, largely due to impaired autophagosome-lysosome fusion, was also noted only in *ttntv-A* but not in *ttntv-Z* models. Moreover, we found that genetic manipulation of *ulk1a* restored autophagy flux and rescued cardiac dysfunction in *ttntv-A* animals. Together, our findings presented adult zebrafish as an in vivo animal model for studying AH in *TTNtv* DCM, demonstrated *TTN* loss of function is sufficient to trigger *ttntv* DCM in zebrafish, and uncovered *ulk1a* as a potential therapeutic target gene for *TTNtv* DCM.

## Abstract 2

The exon-dependent nature of TTNtv cardiomyopathy can be modeled in zebrafish, and repair of autophagy dysregulation in this model ameliorated disease-associated phenotypes.

## Graphical abstract

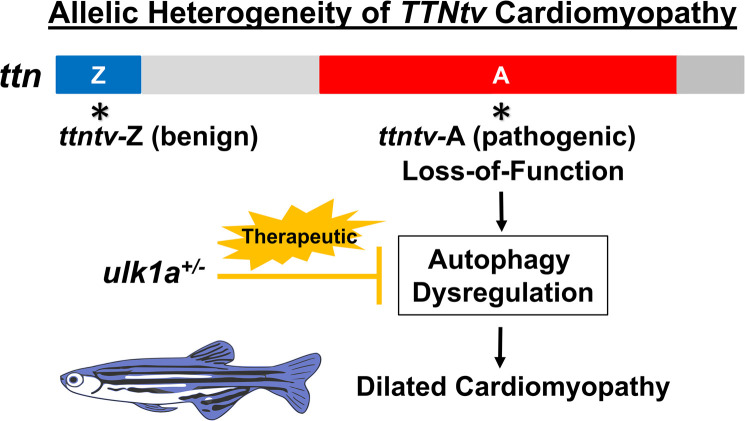

## Introduction

Dilated cardiomyopathy (DCM) is a heart muscle disorder that is characterized by dilation of the ventricle and systolic contractile impairment (1). It is a common cause of heart failure and associated with substantial morbidity, mortality, and health care expenditure (2–4). More than 50 genes have been linked to DCM, and the most predominant genetic factor is truncational variants in the *TITIN* gene (*TTNtv*s) (5–7), accounting for approximately 20% of cases (8–13). However, *TTNtv*s have also been found in about 1% of the reference population, who do not exhibit phenotypes (11, 14, 15). Studies in human patients found that *TTNtv*s affecting exons encoding the C-terminal A-band region (*TTNtv-A*s) are more likely to be pathogenic than those encoding the N-terminal Z-band region (*TTNtv-Z*s) (10, 16, 17). This position-dependent feature of *TTNtv*, termed allelic heterogeneity (AH), is a major hurdle for the implementation of genotype-guided diagnosis and therapy, and the underlying mechanisms remain largely elusive (18–20).

Although *TTNtv* DCM has been successfully modeled in rat, mouse, zebrafish, and induced pluripotent stem cells (iPSCs), AH has only been recapitulated in iPSCs (16, 17). In one iPSC study, it was shown that cardiac microtissue expressing A-band *TTNtv* exhibited less than half the contractile force of those expressing I-band *TTNtv* or WT (16). Similarly, another iPSC study showed that *TTNtv-Z*-cardiomyocytes had sarcomeres and were able to contract whereas *TTNtv-A* cardiomyocytes were not able to (17). In contrast, rat models of *Ttntv-Z* and *Ttntv-A* manifest similar cardiac phenotypes — normal under resting conditions while higher strain rates and higher left ventricle pressures develop under transaortic constriction stress (21). In mice, a *Ttntv-A* model harboring a 2 bp insertion in an A-band exon was established to mimic a human pathogenic variant (22, 23). Heterozygous mutants showed left ventricular dilation, impaired fractional shortening, and diffused myocardial fibrosis when stressed with angiotensin II or isoproterenol (24). No mouse *Ttntv-Z* model has yet been reported. Several other mouse mutants that contain internal deletions of important *Ttn* domains, such as N2B, PEVK, I-band/A-band junction, and kinase domains, or a missense mutation, exhibit cardiomyopathy-like phenotypes (25–30), but they are not *TTNtv*s. In zebrafish, the first *ttntv* mutant, *pickwick*, contains a nonsense mutation in the N2B domain and is considered an embryonic *ttntv* model (31); an adult zebrafish *ttntv-A* mutant was recently reported to develop a DCM-like phenotype (32). We previously generated a panel of *ttntv-A* and *ttntv-Z* mutants and revealed AH during somitogenesis (33); however, whether these mutants recapitulate AH of *TTNtv* DCM is unknown.

Three hypotheses have been proposed to explain the AH of *TTNtv* DCM. First, the haploinsufficiency/exon usage hypothesis argues that *TTNtv-Z*s have less effect on the protein levels of full-length *TTN* than *TTNtv-A*s. This hypothesis is supported by studies directly correlating exon usage with pathogenic mutations in patients with DCM (13) and engineered iPSCs (16) and indirectly by the exon-dependent penetrance of *ttntvs* in zebrafish somitogenesis (33). Second, the Cronos hypothesis predicts that disruption of the Cronos isoform, a shorter *TTN* isoform that shares the C-terminal 124 exons with the full-length *TTN* (34), predisposes animals to the development of *TTNtv* DCM. This hypothesis was initially proposed based on findings in zebrafish somitogenesis, in which *TTNtv-A*s but not *TTNtv-Z*s disrupted the Cronos isoform (34, 35), and was later supported by a study in iPSCs (17). Third, the toxic peptide hypothesis proposes that *TTNtv*s in the A-band result in truncated *TTN* peptides that exert dominant toxic effects (10). This idea has attracted more attention recently because truncated *TTN* peptides have been detected in human patients with *TTNtv* DCM (36, 37). Whether these hypotheses are mutually exclusive or coexist remains to be elucidated.

The establishment of *TTNtv* DCM animal models laid the foundation for searching for dysregulated signaling pathways (38–41). A recent study in a rat *Ttntv* model uncovered autophagy dysregulation, i.e., increased expression of LC3-II and p62 proteins at steady state and failure to further accumulate LC3-II upon administration of the lysosome inhibitor bafilomycin A1 (BafA1) in Ttn-knockdown cells (21). It remains unclear whether autophagy can be leveraged for therapeutic benefits (42).

Here, using a collection of zebrafish *ttntv-A*s and *ttntv-Z*s mutants, we showed that AH can be modeled in *ttntv* homozygous embryos as well as heterozygous adult fish. We generated null mutants with deletion of the full-length *ttn* genes and revealed a loss-of-function nature of *TTNtv* DCM. Finally, we demonstrated that autophagy is differentially dysregulated in *ttntv-A* versus *ttntv-Z* mutants and that targeting *ulk1* could be a viable therapeutic avenue.

## Results

### Cardiac function and sarcomere structure are more severely affected in homozygous ttntv-A embryos than in ttntv-Z mutants.

**Figure 1:**
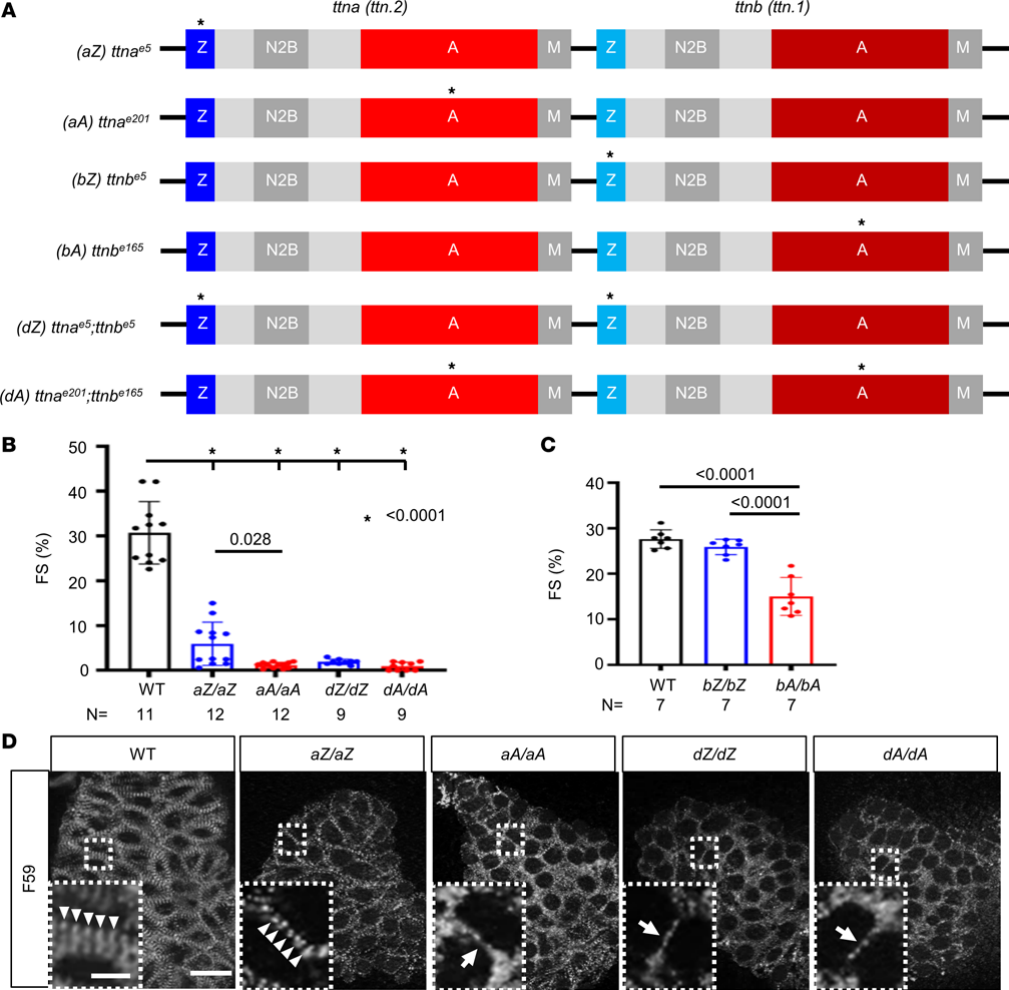
AH in cardiac function and sarcomere structure is noted in homozygous *ttntv* embryos. (**A**) Schematics of the *ttntv* mutants. These mutants have been generated previously (33). * indicates the location of truncation mutations. (**B**) Quantification of fractional shortening in the embryonic hearts at 2 dpf. *P < 0.0001. (**C**) Quantification of fractional shortening in the embryonic hearts at 9 dpf. (**D**) Comparison of cardiac sarcomere structure in 2 dpf embryos via F59 antibody staining. Scale bar = 20 μm. Insets show enlarged images of the area enclosed with dashed lines. Scale bar = 5 μm. Arrowheads indicate sarcomeres with striated patterns. Arrows indicate immature sarcomeres with less striated patterns.

Zebrafish have 2 homologs of mammalian *TTN*, namely, *ttna* (also designated *ttn.2*) and *ttnb* (also designated *ttn.1*). They are in tandem on chromosome 9, with *ttna* being predominantly expressed in the heart (43). Prompted by the success of modeling AH during somitogenesis (32), we decided to analyze the cardiac phenotypes of these *ttntv-A* and *ttntv-Z* embryos (Figure 1A). Comparing *aZ/aZ* with *aA/aA* mutants that affect *ttna*, we noted that approximately 48% of *aZ/aZ* fish maintained weak heart contraction while 98% of *aA/aA* mutants manifested complete or near-complete cessation of contraction at 48 hpf (Table 1 and Figure 1B). The residual contraction of the ventricle in the *aA/aA* mutants appeared to be a passive movement initiated from the contraction in the atrium (Supplemental Video 1; supplemental material available online with this article; https://doi.org/10.1172/jci.insight.175501DS1). Moreover, *ttnb* mutants were generally normal until 9 dpf, when reduced cardiac contraction was noted in *bA/bA* but not *bZ/bZ* (Figure 1C).

Visual inspection of the *ttntv* embryos indicated pericardial effusion, defective somite structure (Supplemental Figure 1, A and C), and abnormal ventricle size (Supplemental Figure 1, D and F) in *aA/aA*, *aZ/aZ*, *dA/dA*, and *dZ/dZ* but not *bA/bA* or *bZ/bZ*. We compared the sarcomere structure between *ttntv-Z* and *ttntv-A* mutants by immunostaining with F59, an anti–myosin heavy chain antibody that labels the thick filament (44). In agreement with the lack of contraction, the striation of the myofibrils was severely disrupted, and the lateral growth of sarcomeres was halted in homozygous *aA/aA* mutants (Figure 1D). In contrast, striated sarcomeric structures were still detectable in approximately 50% of *aZ/aZ* mutants, consistent with half of the *aZ/aZ* mutants retaining certain contractions (Figure 1, B and D). Although *bZ/bZ* exhibited sarcomeric defects in somites at 9 dpf, as reported previously (Supplemental Figure 2A) (33), we did not observe any sarcomere defects in their hearts (Supplemental Figure 2B). Furthermore, we compared *dZ/dZ* and *dA/dA* double mutants that affected both *ttna* and *ttnb* in the corresponding exons; however, this comparison was not informative for AH because both mutants had silent hearts and lacked striated myofibrils (Table 1 and Figure 1, B and D).

### Cardiomyopathy-like phenotypes are noted in heterozygous zebrafish ttntv-A adult mutants but not in ttntv-Z mutants.

**Figure 2:**
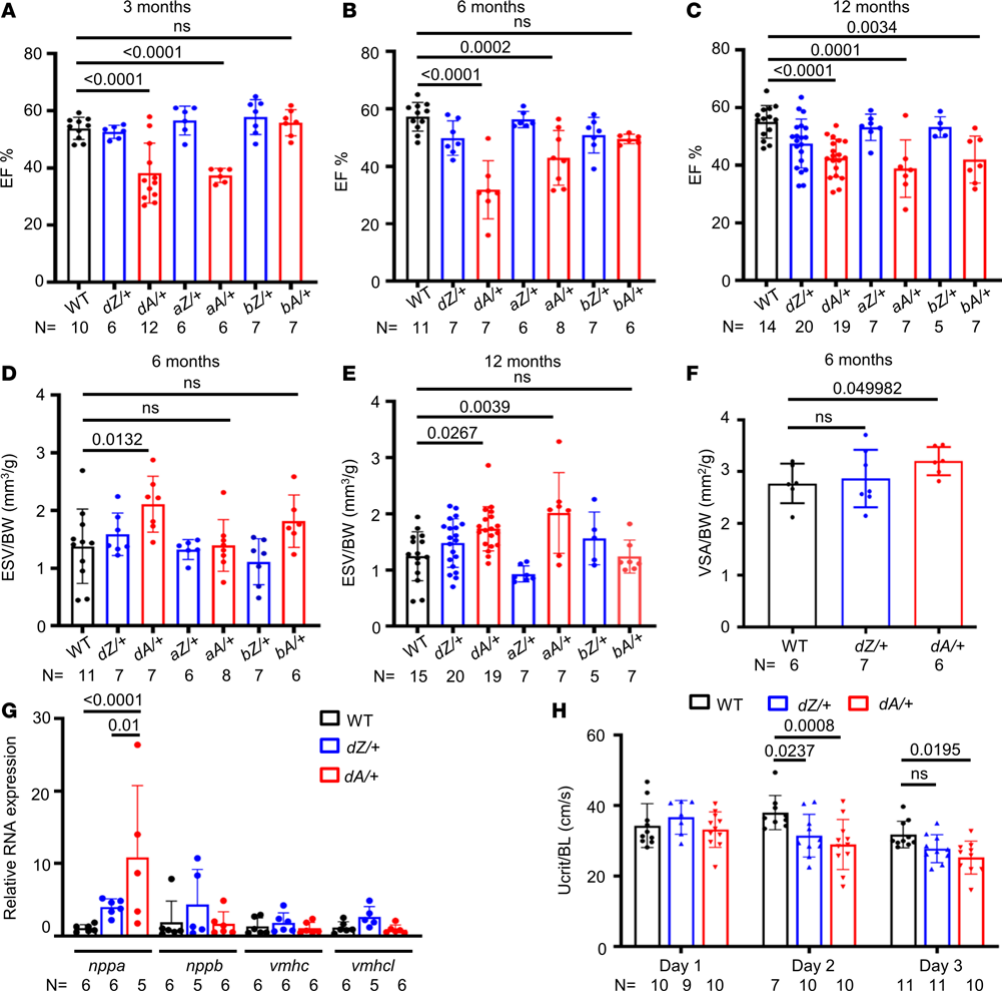
AH in cardiac contraction is noted in adult zebrafish heterozygous *ttntv* mutants. (**A**–**C**) Quantification of the ejection fraction in adult zebrafish at 3–12 months via high-frequency echocardiography. One-way ANOVA was applied. (**D** and **E**) Quantification of end-systolic volume/body weight (ESV/BW) in adult zebrafish at 6–12 months. One-way ANOVA was applied. (**F**) Comparison of dissected ventricular size in 6-month fish. Independent samples *t* test was applied to compare WT with *dZ/+* and WT with *dA/+*. (**G**) Expression of *nppa*, *nppb*, *vmhc*, and *vmhcl* gene transcripts by quantitative real-time PCR in 6-month WT, *dZ/+*, and *dA/+* fish. One-way ANOVA was applied. (**H**) The swimming challenge test indicated a reduced swimming ability in *ttntv* fish compared with WT fish at 15 months of age. Two-way ANOVA was applied to compare WT versus *dZ/+*, WT versus *dA/+*, and *dZ/+* versus *dA/+* in each day group. Data are presented as mean ± SD. VSA, ventricular surface area; BL, body length.

To better understand patients with *TTNtv*, we examined heterozygous *TTNtv-A* and *TTNtv-Z* mutants at the adult stage. Using high-frequency echo to quantify cardiac pump function, we found a significant reduction in ejection fraction (EF) in *aA/+* but not *aZ/+* fish starting from the age of 3 months (Figure 2, A–C). Similar cardiac dysfunction was also noted in *dA/+* but not *dZ/+* mutants (Figure 2, A–C). We did not detect significant changes in markers for mitochondrial function and mass (Drp1 and Tom20) in the hearts of 3-month *dA/+* fish (Supplemental Figure 3), suggesting that mitochondria dysfunction is not prominent at this early pathological stage. While cardiac dysfunction was not detected in *bA/+* fish until 12 months, *bZ/+* fish remained normal (Figure 2, A–C). Moreover, mirroring a recent report, cardiac function was more significantly reduced in female fish than in male fish (Supplemental Figure 4) (32).

To further define DCM-like phenotypes in *ttntv-A*s, we carried out detailed analysis of the echo data and found that the end-systolic volume (ESV) was significantly increased in *dA/+* and *aA/+* fish (Figure 2, D and E). At 6 months of age, standardized ventricle surface area was enlarged in *dA/+* but not *dZ/+* fish (Figure 2F). In contrast with the myocardium of rodents and humans, the myocardium of the adult zebrafish is composed of a compact layer and a trabeculated layer. Because a clearly defined wall is absent, the density of the trabeculated myocardial muscles has been utilized as a metric to assess cardiomyopathy-like changes in a zebrafish heart (45, 46). No significant changes of the muscle density were found in the zebrafish *ttntv* model at 3 months of age (Supplemental Figure 5, A and B). At the mRNA level, the cardiac remodeling marker *nppa* was greatly activated in 6-month-old *dA/+* mutants and, to a lesser extent, *dZ/+* mutants (Figure 2G), suggesting some abnormality in *dZ/+* as well. Given that patients with DCM perform poorly on physical activity assessments, we assessed the exercise capacity of the *ttntv*s via a continuous 3-day swimming test. As widely accepted, Ucrit, instead of total distance, is a recognized index to measure swimming capacity (47, 48). The assay is always carried under the same light condition (49). We found lower Ucrit in both *dA/+* and *dZ/+* at day 2 but only in *dA/+* at day 3 (Figure 2H), supporting the notion that *dZ/+* is more resilient but not entirely normal.

**Figure 3:**
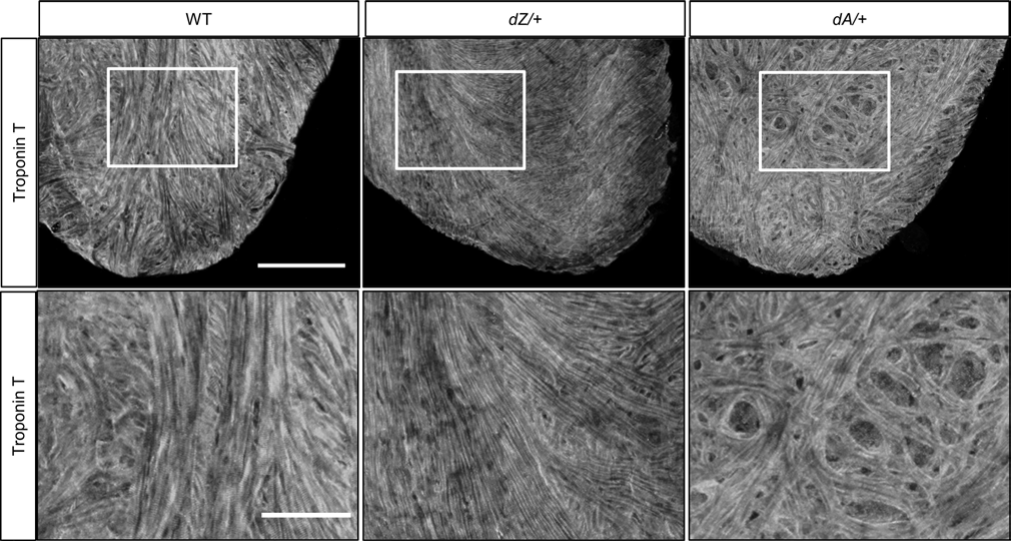
AH in myofibril organization is noted in adult zebrafish *ttntv* mutants. Shown is anti–Troponin T immunostaining of a whole heart isolated from a 15-month-old zebrafish using tissue clearance technology. Distorted myofibrils were noted in *dA/+* but not in *dZ/+* and WT. *n* = 3 in each group. Scale bar = 100 μm. Images in the bottom panel are enlarged images of the marked area in the upper panel. Scale bar = 50 μm.

To assess striated sarcomere structure in myofibrils, we developed a tissue-clearing whole-mount staining protocol that enables better resolution of the sarcomeric structure in isolated adult fish whole hearts. While the striated pattern of sarcomeres was not affected, we noted aberrant trabecular muscle organization in the hearts of *dA/+* but not *dZ/+* fish at 15 months. In contrast with well-aligned myofibrils, some trabecular muscles in *dA/+* formed unique circular structures (Figure 3). Together, these data strongly suggested that *ttntv*s in zebrafish recapitulate the AH of *TTNtv* DCM in humans.

### Loss of function of ttn mutants manifests DCM-like phenotypes.

**Figure 4:**
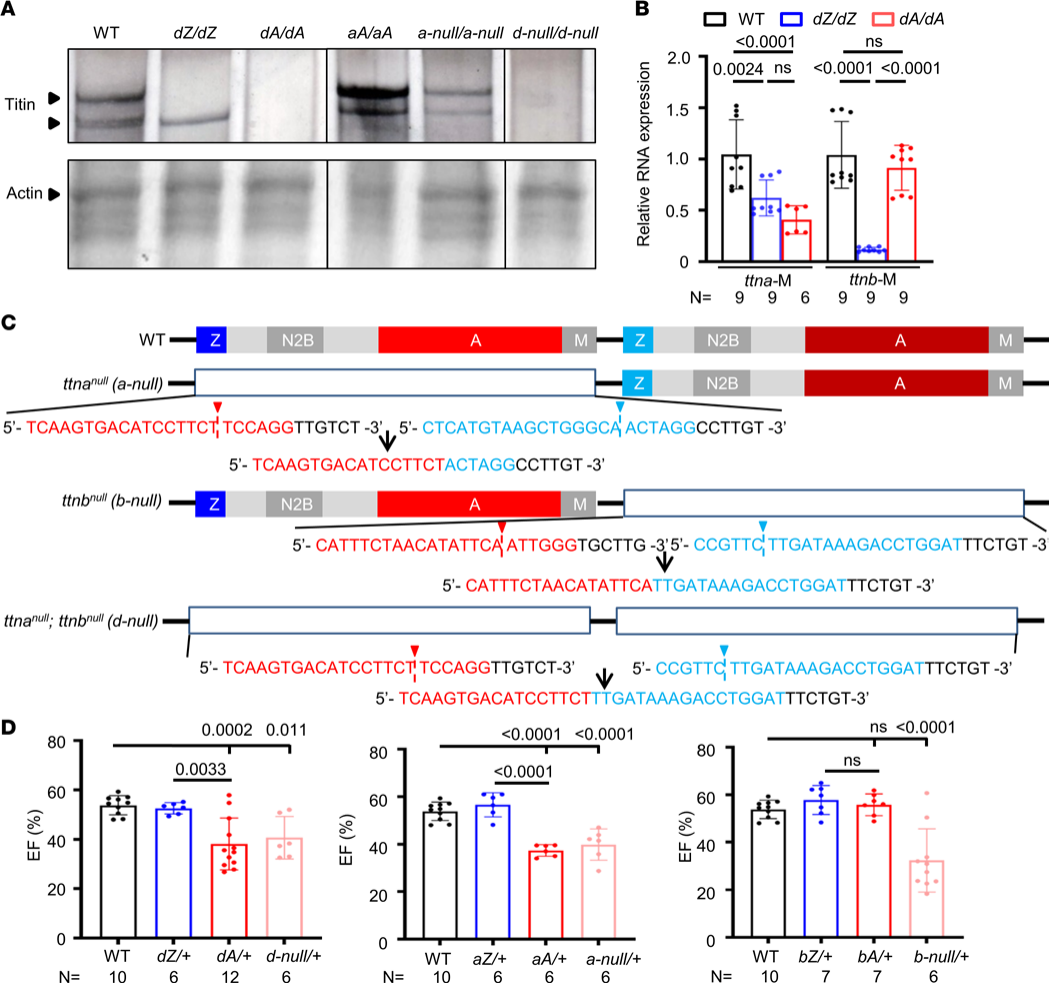
*ttn-null* mutants manifest similar phenotypes to *ttntv-A* but not *ttntv-Z* mutants. (**A**) Protein analysis of 2 dpf larvae of WT, *dZ/dZ*, *dA/dA*, *aA/aA*, *a-null/a-null*, and *d-null/d-null* mutants. The upper lane shows silver staining of a 2% SDS-agarose gel. The same protein lysates were then analyzed by Coomassie blue staining with a 12.5% SDS-PAGE system, as shown in the bottom lane. The expression of Actin was used as a loading control. (**B**) *ttna* and *ttnb* mRNA expression levels in *dA/dA* and *dZ/dZ* embryos, as revealed by quantitative PCR using primer pairs that recognize the M-line exons of *ttna* and *ttnb*, respectively. (**C**) Schematics of the location of sgRNAs that were used to generate the large deletions in *ttn-a-null*, *ttn-b-null*, and *ttn-d-null*. The red and blue sequences are sgRNAs on either side. Arrowheads indicate the cutting sites, and the resulting genome sequence is listed below. (**D**) High-frequency echocardiography was performed in adult zebrafish at 6 months to quantify the ejection fraction. One-way ANOVA was used to compare multiple groups for each mutation. Data are presented as mean ± SD.

To investigate the molecular mechanisms underlying AH in *TTNtv* DCM, we examined Titin protein expression using agarose-based SDS protein gels (50). Consistent with previous results (33), *dZ/dZ* mutants lost the higher–molecular weight Titin band but maintained the lower–molecular weight Titin band (Figure 4A), suggesting again the affected exons are unnecessary for some Titin isoforms. Surprisingly, unlike previous F_2_ generation mutants (33), *dA/dA* fish in the current F_8_ generation no longer expressed any full-length Titin proteins (Figure 4A). To assess whether nonsense-mediated RNA decay was aggravated from the F_2_ through the F_8_ generation, we examined mRNA expression. We found that the changes in transcript levels in the F_8_ generation were the same as those in the F_2_ generation (33), i.e., fewer *ttna* transcripts in *dA/dA* than in *dZ/dZ* and undisturbed *ttnb* transcripts in *dA/dA* (Figure 4B), suggesting that the depletion of the full-length Titin protein in the F_8_ generation occurs at the protein level rather than the transcription level. Of note, truncated Titin proteins were not detected in either mutant (data not shown).

To ensure that AH indeed results from loss of function of full-length Titin, we generated null mutants with the entire genomic sequences of *ttna*, *ttnb*, or both removed via CRISPR-mediated genome editing (Figure 4C). As expected, both Titin bands were reduced in *a-null/a-null* fish and absent in *d-null/d-null* mutants (Figure 4D). In the somites, sarcomere assembly was halted at the premyofibril stage, as manifested by the greatly reduced width of the Z-disc and length of the sarcomere in *d-null/d-null* mutants (Supplemental Figure 6, A–C). Similar sarcomeric defects were noted in the heart (Supplemental Figure 6D), pericardial effusion and abnormal ventricle could be detected (Supplemental Figure 1, B and E), and cardiac contraction was nearly completely halted in *d-null/d-null* mutants (Supplemental Video 1). We raised the heterozygous null mutants to adulthood and found that cardiac functions in *d-null/+* and *a-null/+* were significantly impaired at 6 months, comparable to that seen in *dA/+* and *aA/+* (Figure 4E). Interestingly, *b-null/+* fish exhibited an earlier decline in cardiac function (6 months) (Figure 4D) than *bA/+* fish (12 months) (Figure 2, B and C). Together, these data strongly suggested a loss-of-function nature of *ttntv* DCM in adult zebrafish models.

### Defective autophagic flux is noted in ttntv-As but not ttntv-Zs.

**Figure 5:**
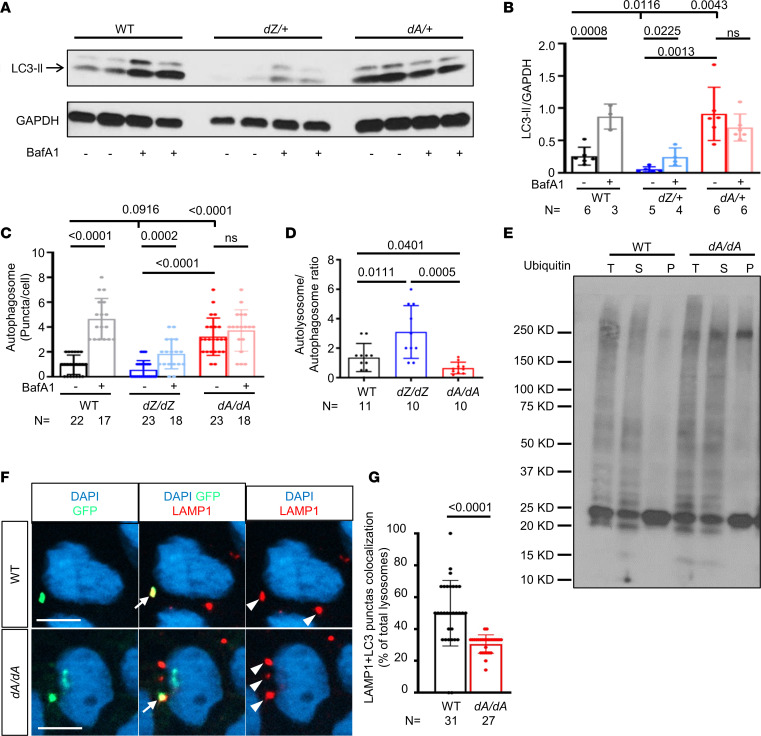
AH in autophagy dysregulation is noted in *ttntv* models, and autophagic flux is affected by *ttntv-A*. (**A**) Representative Western blot of LC3-II from 12-month-old zebrafish hearts. (**B**) Quantification of **A**; autophagy is differentially dysregulated in *dA/+* vs. *dZ/+* fish, with *dA/+* showing increased basal LC3-II and impaired autophagic flux, while *dZ/+* exhibits reduced basal LC3-II. (**C**) Quantification of autophagosome puncta in 2 dpf BafA1-treated embryos (Supplemental Figure 7A). (**D**) Quantification of the autolysosome/autophagosome ratio of 2 dpf non-BafA1-treated embryos (Supplemental Figure 7A). (**E**) Ubiquitin-conjugated (Ub-conjugated) proteins were moderately increased in detergent-insoluble fractions of *dA/dA* embryo lysates. The *dA/dA* fish and WT siblings at 5 dpf were subjected to cell fractionation, and the resulting total lysates (T), soluble fractions (S), and insoluble fractions (P) were analyzed by Western blot using an Ub antibody. The blot is representative of 3 independent experiments. (**F**) Representative image and quantification of LC3-GFP and lysosome marker LAMP1 colocalization compared with the total LAMP1-stained lysosome. The arrow indicates a yellow puncta with both GFP and LAMP1 that represents colocalization, and the arrowhead indicates a red puncta with only LAMP1. Scale bar: 5 μm. (**G**) Quantification of **F**. In this figure, independent samples *t* test is used. Data are presented as mean ± SD.

**Figure 6:**
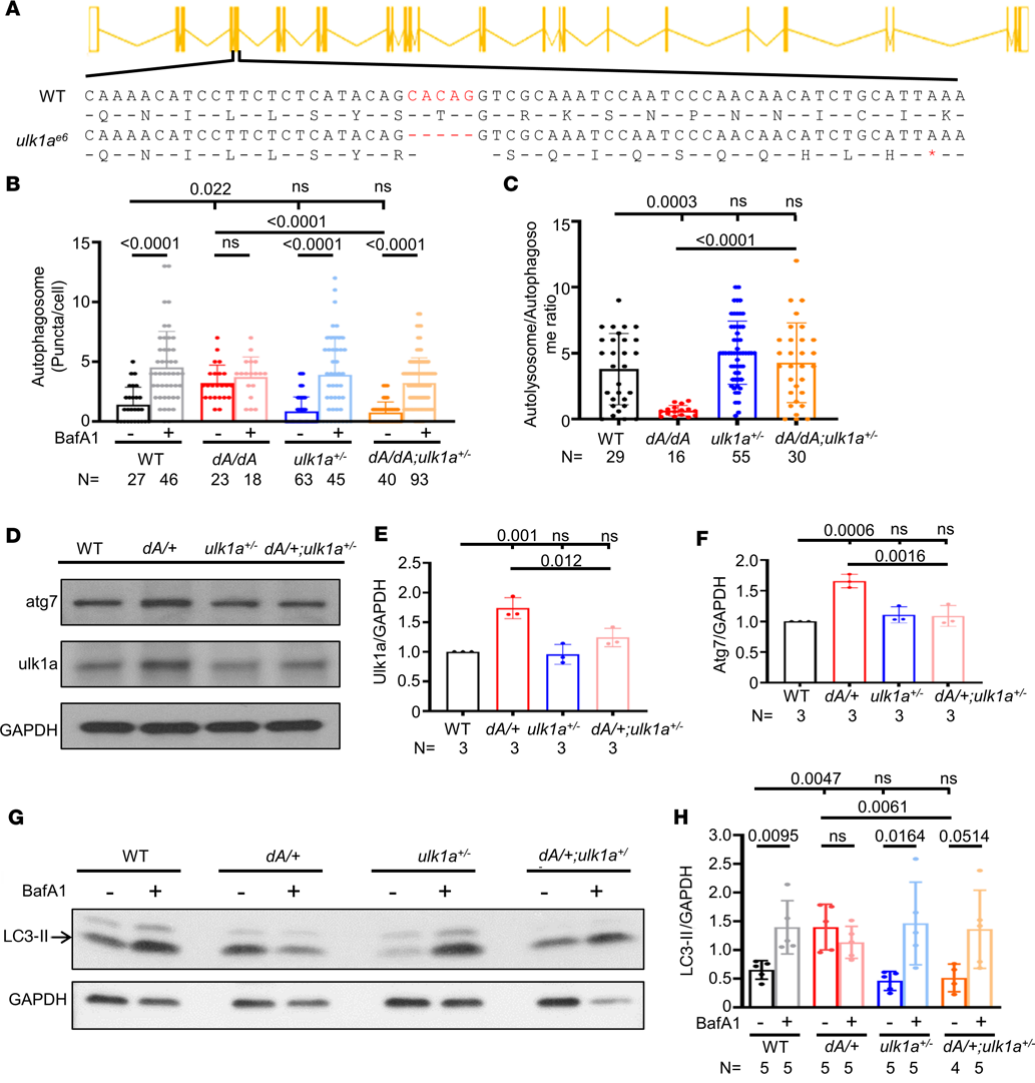
*ulk1a* mutants repair defective autophagic flux in both embryonic and adult stages of *ttntv-A* mutants. (**A**) Schematics of *ulk1a^e6^* generated by MMEJ sgRNA. Red indicates deleted nucleotides. * indicates the nonsense codons. (**B** and **C**) Quantification of the autophagosome puncta in 2 dpf embryos either with or without BafA1 treatment and the autolysosome/autophagosome ratio of BafA1-untreated groups. *ulk1a*^+/–^ can reduce the basal autophagosome level of *dA/dA* and can recover the autophagic flux affected by *dA/dA*. One-way ANOVA was applied. (**D**–**F**) Representative Western blot and quantification of ulk1a and atg7 from 3-month zebrafish hearts. One-way ANOVA was applied. (**G** and **H**) Representative Western blot and quantification of LC3-II from zebrafish hearts either with or without BafA1 treatment at 3 months. Independent samples *t* test was applied to compare 2 groups. Data are presented as mean ± SD.

Because dysregulated autophagy has been reported to be a pathological event in a rat *Ttntv* model (21), we asked whether dysregulated autophagy also occurs in zebrafish *ttntv* models. Similar to the rat model, we noted significantly increased basal LC3-II expression in 12-month-old *dA/+* mutants that failed to further accumulate upon treatment with BafA1, an inhibitor of autophagosome-lysosome fusion (Figure 5, A and B), suggesting a defect in autophagic flux. This autophagic dysregulation can be detected in *dA/+* mutants as early as 3 months (*P* < 0.01, see Figure 6, G and H). In contrast, *dZ/+* hearts manifested a different mode of autophagic dysregulation: a normal response to BafA1 treatment despite reduced basal LC3-II levels (Figure 5, A and B).

To visually assess autophagy dysregulation at the cellular level, we injected the *mCherry-EGFP-LC3-II* tandem repeat plasmid into embryos at the 1-cell stage for fluorescence analysis of puncta. The hearts were dissected at 2 dpf and treated with either BafA1 or DMSO solution in a culture dish, and then autophagosomes (yellow dot) and autolysosomes (red dot) were quantified (Supplemental Figure 7A). We found significantly more autophagosomes in *dA/dA* hearts that could not be further increased in response to BafA1 treatment, as well as fewer (though not significantly, *P* = 0.0916) autophagosomes in *dZ/dZ* fish that were responsive to BafA1 (Figure 5, C and D). Consistent with the flux defect in *dA/dA* fish, ubiquitin-tagged protein aggregates in the detergent-insoluble portion, which is usually degraded via the autophagy pathway, accumulated (Figure 5E). Next, we costained LC3-GFP (autophagosome marker) and LAMP1 (lysosome marker) and found a reduced colocalization in *dA/dA* hearts (Figure 5, F and G) (51), indicating an impairment in autophagosome-lysosome fusion. In *d-null/d-null* mutants, we also noted aberrant accumulation of autophagosome and defective autophagic flux (Supplemental Figure 7, B and C), as has been noted in *dA/dA*, supporting a loss-of-function nature underlying how *ttntv* mutants result in autophagic defects. Together, these data strongly suggested that AH in autophagy dysregulation could occur in both zebrafish heterozygous adults and homozygous *ttntv* embryos.

### ulk1a can be targeted to repair defects in autophagic flux and ameliorates cardiomyopathy-like phenotypes in ttntv-As.

To determine whether defective autophagy can be repaired for therapeutic benefits, we elected to knock down *atg7* that is involved in autophagosome formation and *ulk1a* that functions in both autophagosome formation and autophagosome-lysosome fusion (52–54) via microhomology-mediated end-joining (MMEJ) technology (55, 56). At the average knockout (KO) efficiency approximately 80% (Supplemental Table 2), injection of MMEJ-inducing sgRNAs against *ulk1a* or *atg7* did not cause significant autophagic changes in WT fish (Supplemental Figure 7A). However, knockdown of Ulk1a but not Atg7 was able to normalize steady-state autophagosome numbers and restore response to BafA1 treatment in *dA/dA* hearts (Supplemental Figure 7A). At the cellular level, injection of *ulk1a*-sgRNA improved LC3-GFP and LAMP1 colocalization in the *dA/dA* fish (Supplemental Figure 7, B and C), indicating a repair in the autophagosome-lysosome fusion.

To confirm this observation from transient genetic analysis, we generated a stable *ulk1a* mutant that contains a 5 bp deletion in exon 6 of the *ulk1a* gene (Figure 6A). *ulk1a* transcripts were significantly reduced in the *ulk1a^+/–^* mutant (Supplemental Figure 9), presumably due to nonsense-mediated RNA decay. Consistent with our observation from transient genetic assay, both *ulk1a^+/–^* and *ulk1a^–/–^* were able to rescue defects in the autophagic flux in the *dA/dA* mutant (Figure 6, B and C, and Supplemental Figure 10, A and B). In adult *dA/+* mutant hearts, expression levels of both Atg7 and Ulk1a were increased, which could be attenuated by *ulk1a^+/–^* (Figure 6, D–F). Moreover, *ulk1a^+/–^* could restore autophagic flux in adult *dA/+* hearts, as indicated by the normalized LC3-II levels in the basal and BafA1-treated states (Figure 6, G and H).

**Figure 7:**
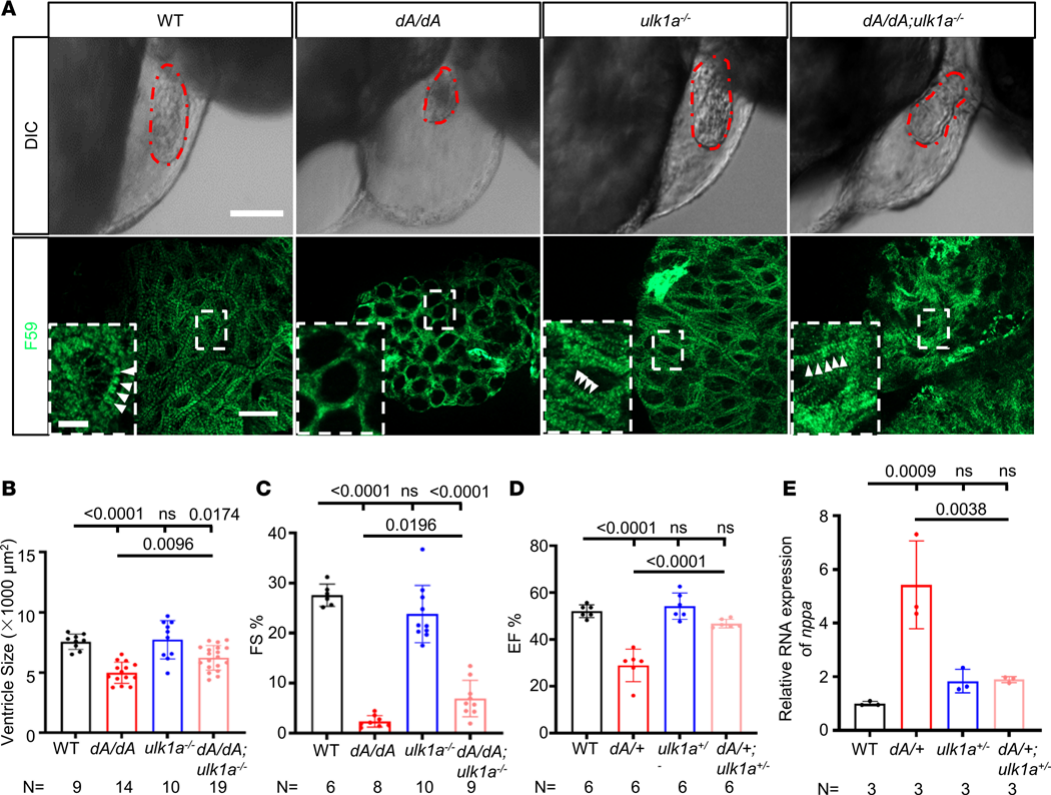
*ulk1a* mutants repair cardiac dysfunction in both adult and embryonic stages of *ttntv-A* mutants. (**A**) Immunostaining and differential interference contrast (DIC) images of 2 dpf embryos. Sarcomere structures were obtained by immunostaining with an anti-F59 (green) antibody in WT, *dA/dA*, *ulk1a^–/–^*, and *dA/dA ulk1a^–/–^*. Higher magnifications of the boxed areas are shown in the insets. White arrowheads indicate striated sarcomere structures. The embryonic ventricle is outlined by dashed red lines in the DIC images. The scale bar of the upper panel is 100 μm, the lower panel is 10 μm, and the inset is 2.5 μm. (**B**) Quantification of the ventricle area based on the area outlined by the dashed red lines in the DIC panels of Figure 7A. (**C**) Quantification of fractional shortening in embryonic hearts. (**D**) High-frequency echocardiography was performed on 6-month-old zebrafish to quantify EF%. (**E**) Evaluation of *nppa* gene transcript expression by quantitative real-time PCR in hearts from 3-month-old adult zebrafish. One-way ANOVA was used to compare multiple groups for each mutation. Data are presented as mean ± SD.

Encouraged by the rescuing effects in autophagy, we examined whether *ulk1a* mutants can ameliorate cardiomyopathy-like phenotypes in *dA*/*dA* embryos and *dA/+* adults. Indeed, we found that cardiac defects, including the distorted shape of the ventricle (Figure 7A), reduced ventricular size (Figure 7B), and reduced cardiac function (Figure 7C) in *dA/dA*, could be effectively rescued in the *dA/dA ulk1a^–/–^* double mutants. Consistent with the rescued cardiac contractility, the disrupted sarcomere structure was partially rescued (Figure 7A). At the cellular level, we noted unchanged cardiomyocyte size in the outer curvature and increased nucleus size in *dA/dA*, the latter of which could be partially rescued in *dA/dA ulk1a^–/–^* double mutants (Supplemental Figure 11). Moreover, cardiomyopathy-like phenotypes in *dA/+* could be rescued in *dA/+ ulk1a^–/–^* double mutants at 3 months of age (Supplemental Figure 12) and in *dA/+ ulk1a^+/–^* double mutants at 6 months of age (Figure 7D), as indicated by the improved cardiac function. Consistent with these data, aberrant activation of *nppa* could be rescued in *dA/+ ulk1a^+/–^* double mutants (Figure 7E). Together, our data suggested *Ulk1a* as a therapeutic target gene for *TTNtv* DCM.

## Discussion

### AH in TTNtv DCM can be recapitulated in adult zebrafish.

One of the novelties of this manuscript is that we present zebrafish as an in vivo model that is able to recapitulate AH in *TTNtv* DCM. Cardiomyopathy-like phenotypes occur only in adult heterozygous *ttntv-A* mutants such as *dA/+* but not in *ttntv-Z* mutants such as *dZ/+*. AH in cardiac dysfunction was also noted in *aA/+* or *bA/+* but not *aZ/+* or *bZ/+*. Consistent with the lower cardiac expression level of *ttnb* in zebrafish heart, *bA/+* manifests phenotypes at an older age than either *aA/+* or *dA/+*. A limitation of our present study is that we only assessed a single exon that affects either the Z-disc or A-band. Investigations of more *ttntv*s affecting other *ttn* exons are justified in the future, which could potentially establish zebrafish as a faithful model for assessing the pathogenicity of *TTNtv*s suggested from human patients.

We then defined the pathogenesis of *ttntv* DCM in adult zebrafish by detailed phenotyping of the *dA/+* allele. Similar to a previous study of a *ttntv-A* affecting zebrafish *ttna* exon 202 that corresponds to human exon 327 in *TTN* (32), we noted decreased cardiac function, aberrant expression of molecular markers for heart remodeling, myofibril abnormality, and reduced exercise capacity, which are all phenotypic traits of DCM (Figures 2 and 3). The decreased EF percentage could be attributed to increased ESV and ventricular cavity enlargement. Moreover, we detected additional phenotypes in our *dA/+* allele than in the reported *ttna^tv^* allele (32). First, we were able to detect cardiac dysfunction as early as 3 months in *dA/+*, probably because both *ttn* alleles have been mutated and because Ttn proteins are nearly completely eliminated in *dA/dA* at the F_8_ generation (Figure 4A). Second, we used a tissue-clearing technique that allowed us to better define structural changes in the trabecular muscle. We noted normal sarcomere structure at 15 months, when trabecular muscle disarrangement appeared in *dA/+* but not *dZ/+* fish. Given that reduced EF percentage is already detectable as early as 3 months, when mitochondria dysfunction does not occur, and long before trabecular muscle disorganization, our longitudinal observation strongly suggested that neither defective sarcomere structure nor mitochondria dysfunction is a primary pathological event but rather sequential abnormalities of *ttntv* DCM.

### Loss of function of ttn is sufficient to trigger DCM-like phenotypes.

The establishment of zebrafish as an in vivo animal model for AH in *ttntv* DCM enabled us to further pinpoint the underlying mechanisms. We generated null mutants with complete removal of all zebrafish Ttn proteins. Because genetic compensation should have been effectively eliminated, our observation that heterozygous *d-null/+* manifests DCM-like phenotypes provided a strong piece of genetic evidence supporting that *ttn* loss of function is sufficient to trigger DCM-like phenotypes. This genetic evidence is in line with both the haploinsufficiency hypothesis and Cronos hypothesis (11, 17) because both full-length Titin protein and the shorter Titin Cronos isoform are presumably depleted in our null mutants. However, our current data cannot distinguish between the haploinsufficiency hypothesis and the Cronos hypothesis, which would require future genetic studies that target the Cronos isoform.

Our genetic data do not absolutely disprove the toxic peptide hypothesis because we have not purposed to study truncational TTN peptides. In fact, the 3 hypotheses on AH in *TTNtv* DCM are not mutually exclusive because each of these hypotheses concerns different versions of TTN proteins. The haploinsufficiency hypothesis emphasizes the full-length TTN proteins, the toxic peptide hypothesis relates to the truncated TTN peptides, and the Cronos hypothesis emphasizes a particular shorter TTN isoform. While our current evidence favors the haploinsufficiency hypothesis and the Cronos hypothesis, future studies in the in vivo zebrafish model are still warranted to directly test the toxic peptide hypothesis.

### ulk1a is a therapeutic target gene for ttntv DCM in zebrafish.

The discovery of autophagy dysregulation in the rat *Ttntv* model prompted us to test whether this pathological signaling is conserved in our zebrafish *ttntv* models. Indeed, we observed similar autophagic flux defects, manifesting as an increased accumulation of LC3-II and a lack of response to BafA1 (21). Using *dA/dA* homozygous embryos, our data further suggested that the dysregulated autophagy in *ttntv* DCM is characterized by defective degradation, likely owing to reduced fusion between autophagosome and lysosome. Precisely how *ttntv* leads to autophagosome-lysosome fusion defects warrants future studies. Nevertheless, the fact that these autophagy defects are detectable as early as 3 months of age suggests that autophagy dysregulation is a proximal pathological event triggered by *ttntv*, which is different from myofibril structural changes that occur at 15 months of age, a much later pathological stage. Our data in the *d-null/d-null* mutant further suggested that autophagy defects can be triggered by depletion of Ttn proteins, without any contribution from truncated Ttn as a toxic peptide (Supplemental Figure 7, B and C).

Another potentially novel discovery of the present study is to demonstrate that repairing autophagy could be a therapeutic avenue for *TTNtv* DCM. *ulk1a* was identified as a candidate gene that can be manipulated to repair autophagic defects and to rescue cardiac dysfunction. This rescuing effect is manifested at the autophagosome-lysosome fusion step, and whether the more classic function of Ulk1 in autophagy initiation also contributes to the therapeutic benefits remains to be investigated. While Atg7 expression level is aberrantly increased in *dA*/+ that is attenuated in *ulk1^+/–^*, the seemingly negative data from transient genetic studies of atg7 argues against that changes in Atg7 expression might contribute significantly to the therapeutic effect of *ulk1a* inhibition (Figure 6F and Supplemental Figure 8A).

Recent studies showed complex functions of ULK1 in the autophagosome-lysosome fusion via phosphorylation of its downstream targets STX17 and YKT6, 2 components of the SNARE complex essential for autophagosome-lysosome fusion (57–59). While ULK1-dependent phosphorylation of STX17 promotes the fusion process, phosphorylation of YKT6 impairs autolysosome formation. How STX17 and YKT6 are regulated in our *ulk1a* fish warrants further analysis. Moreover, a mouse KO study showed that perinatal inhibition of either *Ulk1* or *Ulk2* alone in the heart unexpectedly activates autophagy while coinhibition of *Ulk1* and *Ulk2* suppresses autophagy, suggesting an Ulk dose-dependent impact on autophagy (60). Whether this dose-dependent response also occurs in zebrafish models (given the existence of *ulk1a*, *ulk1b*, and *ulk2* in zebrafish) needs to be considered in the future when translating Ulk1-based therapy for *ttntv* DCM.

We noted that knocking down of either *ulk1* or *atg7* did not cause significant autophagy changes (Supplemental Figure 7A). It remains to be determined whether this is owing to the haploinsufficient nature of these CRISPants, compensation by family members, or species difference between zebrafish and mammals. Future studies are warranted to further test these possibilities, including studies of stable *atg7*-null fish and *ulk1 ulk2* compound mutants.

### Zebrafish is a promising animal model for developing genotype-guided therapy for TTNtv DCM.

The present study established zebrafish as an important alternative animal model for studying *TTNtv* DCM. Compared with existing rodent models (16, 17, 22, 25–28, 61), zebrafish models of *ttntv* DCM possess remarkable advantages, including notable cardiac phenotypes without the need for any biomechanical stresses and the capacity to recapitulate AH. Our data highlighted zebrafish as an increasingly valuable animal model for studying cardiomyopathies of different etiologies. In the last decade, a panel of cardiomyopathy models have been generated (62), and novel phenotyping tools have been developed in this vertebrate model with a tiny heart, enabling the definition of both common and unique phenotypes in cardiomyopathies of different etiology (63, 64). In searching therapeutic avenues using these disease models, it appears that different models benefit from different therapeutic strategies. For example, although *bag3* DCM (56), *lamp2* hypertrophic cardiomyopathy (65), and anthracycline-induced cardiotoxicity models (66) benefit from *mtor* inhibition, the *RRAGC^S75Y^* knockin model does not but rather benefits from *tfeb* activation in cardiomyocytes (67). Here, we reported *ulk1a* as a candidate therapeutic target gene for the *ttntv* DCM model. Whether *ulk1a* would benefit other genetic types of cardiomyopathies and whether therapeutic avenues discovered from other cardiomyopathies are extendable to *ttntv* DCM remain to be investigated. Findings from zebrafish need to be tested in mammalian models before further translational efforts. It is possible that systematic studies in the efficient zebrafish model would accelerate the implementation of precision medicine for cardiomyopathies of different etiologies.

## Methods

A detailed version of the Methods section is presented in the online Supplemental Methods.

### Sex as a biological variable.

In Supplemental Figure 4 we examined male and female zebrafish, and similar findings are reported for both sexes (32). In other results, sex was not considered as a biological variable.

### Generation of ttn-null mutants.

Large deletions in *ttn-null* mutants were generated by coinjecting 2 MMEJ-based sgRNAs (68). The sgRNA sequences are listed in Figure 4C. Tail fins from F_1_ generation animals were collected for DNA extraction (HotSHOT method) (69), and the DNA lysates were used as templates for PCR to identify fish harboring the desired large deletions. The primer pairs for *ttna-null* are F: 5′ CGCACCAGTTGTTACTGTC 3′, R: 5′ CATAGTCAGTCTGAACACAAGG 3′; the primer pairs for *ttnb-null* are F: 5′ CAGCAAAAATCACTTTATTCTG 3′, R: 5′ CAAAATGGTGCAGAACTTATGG 3′; and the primer pairs for *ttnd-null* are F: 5′ CGCACCAGTTGTTACTGTC 3′, R: 5′ CAAAATGGTGCAGAACTTATGG 3′. Founders containing large deletions in *ttna-null* were identified by searching embryos with a 134 bp PCR product that contains 5′ TCAAGTGACATCCTTCTACTAGGCCTTGT 3; founders containing large deletions in *ttnb-null* were identified by searching embryos with a 304 bp PCR product that contains 5′ CATTTCTAACATATTCATTGATAAAGACCTGGATTTCTGT 3′; and founders containing large deletions in *ttnd-null* were identified by searching embryos with a 256 bp PCR product that contains 5′ TCAAGTGACATCCTTCTTTGATAAAGACCTGGATTTCTGT 3′.

### LC3-II plasmid injection and puncta quantification.

mCherry-EGFP-LC3-II fragments were amplified from the *pBABE-puro-mCherry-EGFP-LC3-II* plasmid (70) using a primer pair (F: 5′ GGGGATCCGCCGGCCGGATCTGCCACCATGG 3′, R: 5′ CGCTCGAGCCACAGGGTCGACTTACACTGAC 3′) and were inserted into the BamHI-XhoI sites of a pENTR1A vector (Addgene plasmid 11813-011), a middle clone plasmid from the Tol2Kit (71). The attL-to-attR recombination reaction was carried out using a 5′ clone, *p5E-*β*actin* (5.3 kb β*-actin* ubiquitous promoter); a middle clone, *pENTR1A-mCherry-EGFP-LC3-II*; and a 3′ clone, p3E-polyA (SV40 late polyA signal), to generate the final β*-actin*: tandem plasmid.

To generate the β*-actin*: tandem plasmid with a final concentration of 33 ng/μL, 33 ng/μL transpose, 0.2 M KCl, and 0.01% phenol red were freshly mixed. Three nanoliters of the mixture solution was injected into 1-cell–stage embryos. Hearts at 2 dpf were dissected and transferred to a slide with Leibovitz’s L-15 medium. The dissected hearts were treated with 167 nM BafA1 (MilliporeSigma, 19148) or DMSO solution for 2 hours at 28°C. Hearts were then fixed in 4% paraformaldehyde for 7 minutes, washed with PBS for 10 minutes, and mounted with mounting medium containing DAPI (Vector Laboratories, H-1200). Images were documented using a Zeiss fluorescence microscope using a 63× lens. To quantify autophagic puncta, red and yellow dots around the DAPI-stained nuclei were counted manually.

### Statistics.

Data were analyzed by Prism 9 (GraphPad Software by Dotmatics). Independent samples *t* test was used to compare differences between 2 groups, while 1-way ANOVA followed by Tukey’s post hoc test was performed to compare the differences among multiple groups. All quantitative data are presented as the mean ± SD. The detailed *P* values and sample size (*N*) represents the animal number (unless otherwise specifically designated as biological replicates) as listed in the graphs. Unless otherwise specifically mentioned, *P* values less than 0.05 were considered significant.

### Study approval.

Zebrafish (*Danio rerio*; WIK strain) were maintained on a 14-hour light/10-hour dark cycle at 28.5°C. All animals were handled with care following the Mayo Clinic Institutional Animal Care and Use Committee (IACUC) and the *Guide for the Care and Use of Laboratory Animals* (National Academies Press, 2011). The Mayo Clinic IACUC–approved protocol number is A3511.

### Data availability.

Values for all data points in graphs are reported in the Supporting Data Values file. Supplemental Methods and Supplemental Figures are presented in the supplemental materials. More detailed information on data that support the findings of this study is available from the corresponding author.

## Author contributions

XX, PZ, and XL conceived the study; PZ, JL, and SI developed methodology; PZ and JL developed software; XL and XX validated data; PZ, JL, SI, XL, and XX performed formal analysis; PZ, JL, and XX investigated; PZ, JL, and FY curated data; JL and XX wrote the original draft; JL, PZ, FY, XL, and XX reviewed and edited the manuscript; XL and XX supervised the study; XX was the project administrator; and XX acquired funding.

## Supplementary Material

Supplemental data

Unedited blot and gel images

Supplemental video 1

Supporting data values

## Figures and Tables

**Table 1:**
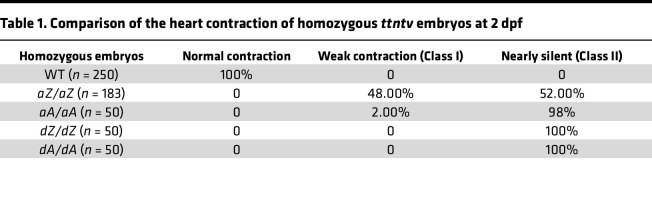
Comparison of the heart contraction of homozygous *ttntv* embryos at 2 dpf

## Version 1 02/27/2024

In-Press Preview

## Version 2 04/08/2024

Electronic publication
