# Supplementary material for: Allelic heterogeneity of TTNtv dilated cardiomyopathy can be modeled in adult zebrafish
Source: JCI Insight. 2024 Feb 27;9(7):e175501. doi: 10.1172/jci.insight.175501 (PMC11128207; doi:10.1172/jci.insight.175501)
Supplement: Supplemental data [file jciinsight-9-175501-s034.pdf]

SUPPLEMENTAL MATERIAL

Expanded Materials & Methods

Study design

In this study, we provide genetic evidence to demonstrate that AH of *TTNtv* DCM can be recapitulated in both embryonic and adult zebrafish in vivo models. And loss-of-function of *ttn* is sufficient to trigger DCM-like phenotypes while *ulk1a* inhibition is a therapeutic avenue. We use similar age and similar body weight *ttn* mutant lines of zebrafish to compare the DCM-like phenotypes, male and female fish are equally included in the research and randomly allocated into different groups, and WT fish are obtained from the genotyping of *ttn* mutant in-crossing lines. Fish with unhealthy conditions or slim bodies are excluded from the analysis. Power analysis was performed by using PASS 11 software to determine approximate sample sizes for the animal heart function analysis(1). For all animal experiments, the animal groups were randomized, and the researchers were blind to the genotypes and treatments of animals.

Quantification of cardiac function via video imaging at the embryonic stage

Zebrafish larvae at the embryonic 2 days post-fertilization (dpf) stage was anesthetized with 0.02% tricaine (Argent Chemical Laboratories) for 2 minutes, placed lateral side up, and held in place with 3% methylcellulose (Sigma-Aldrich). The beating hearts were documented by using a Zeiss Axioplan 2 microscope with a differential interference camera (DIC) lens at 10X magnification, and video clips were

then used to calculate the heart rates and fraction shortening (FS) using the formula [FS=(Ld−Ls)/Ld]. FS was analyzed by using ImageJ software. Ld and Ls represent the length of the short axis of the ventricle at the end-diastolic stage and end-systolic stage, respectively.(2)

Immunofluorescence staining of embryonic hearts and somites

Embryonic hearts at 2 dpf were dissected by using two BD U-100 insulin syringes and were transferred to slides with a 10 μl pipette.(3) Dissected heart tissues and the whole larvae bodies were immediately fixed with 4% PBS-buffered formaldehyde (PFA) for 20 min at room temperature, permeabilized with 0.5% Triton X-100 in PBS (PBST), blocked with 10% normal sheep serum/ PBST for 1 h, and incubated with primary antibodies [anti-myosin heavy chain (1:50, DSHB, F59), anti-β-catenin (1:200, Sigma, #C7207), anti-MEF2(A+C) (1:200, Abcam, #197070)], anti-α-actinin(1:200, Sigma #A7811), anti-GFP(1:200, Abcam, #13970), anti-LAMP1(1:200, Cell Signaling Technology, #15665) at 4℃ overnight and then wash in PBST for 3 times. The hearts were then incubated with secondary antibodies [(Alexa Fluor anti-mouse 488, 1:400; Invitrogen), (Alexa Fluor anti-rabbit 568, 1:400; Invitrogen)] by shaking at RT for 30 mins, washed with PBST 3 times at room temperature, and transferred to a slide that was pre-applied with a mounting medium with DAPI (Vector, H-1200). Slides were covered and images were taken by using a Zeiss Axioplan 2 microscope equipped with Apotome (Carl Zeiss).

Quantification of cardiac function using echocardiography in adult zebrafish

Adult fish cardiac function was measured and analyzed by using a Vevo 3100 imaging system equipped with a 50 MHz linear array sensor (Fujifilm Visual Sonics). Gel (Aquasonic® 100, Parker Laboratories) was spread evenly over the contact surface of the transducer to provide adequate coupling to the zebrafish ventral plane. Adult zebrafish were anesthetized with tricaine (0.02%) for 5 minutes and placed ventral side up in a homemade grooved sponge for fixation. A 50 MHz (MX700) transducer was placed over the ventral side of the zebrafish and slowly panned cephalad to capture an image of the sagittal imaging plane of the heart. B-mode images are acquired by capturing images at the borders of the heart where the sharpest is maximized. Quantification of images was performed using VevoLAB workstation. Acquire and process data as described in a recent report.(4) Cardiac function is quantified by calculating ejection fraction (EF) [EF=(EDV-ESV)/EDV] or fractional shortening (FS) [FS=(Ld-Ls)/Ld], where EDV and ESV are end-diastolic and end-systole ventricular volume, respectively. Ventricular dimensions were normalized from B-mode images using body weights, i.e. EDV/body weight (BW) and ESV/BW.(5)

Quantitative RT-PCR

Ventricles of heart tissues from adult zebrafish were harvested, tissues were homogenized using a Bullet Blender tissue homogenizer (Next Advance Inc.), and total RNA was extracted using TRIzol (Sigma-Aldrich) according to the manufacturer’s instructions. 500 ng isolated total RNA was used to generate cDNAs using the Superscript III First-Strand Synthesis System (Invitrogen). Quantitative RT-

PCR was carried out in 96-well qPCR plates (Applied Biosystems). qPCR primer pairs (*nppa, nppb, vmhcl, vmhc, ulk1a, ulk1b*) are listed in Table S1. Gene expression was normalized to the expression level of *gapdh* using $2^{-\Delta\Delta Ct}$ (cycle threshold) values. Each measurement contained at least three biological replicates.

Swimming tunnel assay

The swimming capacity of adult fish was analyzed by using our reported protocol,(6) which was derived from previous reports.(7, 8) All fish were fasted for 24 hours prior to the first swimming ability measurement. Groups of 10 adult fishes were placed into a swimming tunnel spirometer (Mini Swim 170, Loligo Systems) with an initial water velocity of 9 cm/s and acclimation for 20 min. The water flow was then increased in stages at a rate of 8.66 cm/sec (Ti) every 150 seconds (Tii) until all fish were exhausted and pressed against the baffle at the end of the tunnel by the current. Record the water flow velocities for the final and penultimate stages, Ui and Uii, respectively. The formula for critical swimming ability (Ucrit) is as follows: Ucrit=Ui+[Uii×(Ti/Tii)].(9) Ucrit was then normalized to the body length (BL). The same batch of fish was tested after 1 and 2 days for validation.

Histological staining of adult hearts

All zebrafish heart tissues were harvested from 3-month adult fish under a Leica microscope. After fish were euthanized by 0.05% tricaine for 10 min. The whole hearts were dissected and were immediately fixed with 4% PFA and sent to the Mayo Clinic Histology Core Facility for subsequent sample procession and paraffin

embedding. All histology experiments were performed using paraffin sections cut on a microtome (Leica) and pre-heated on 37 ℃ overnight. Hematoxylin and eosin (H&E) staining was then processed under standardized procedure. The density of the trabecular muscle was quantified using ImageJ software.

Optical Clearing of Adult Zebrafish Heart and Whole-Mount Immunostaining

After fixation and permeabilization, the hearts were cleared with X-CLARITY (Logos Biosystems) according to the manufacturer's instructions.(10) Briefly, the hearts were incubated in hydrogel mixture [0.0025 g X-CLARITY Polymerization Initiator / 1 ml X-CLARITY Hydrogel Solution] overnight at 4°C, transferred to the X-CLARITY™ Polymerization System (Logos Biosystems C20001) with pre-setting vacuum -90 kpa, 37 ℃ for 3 hours, and was placed on a 37°C shaker for 3 days with tissue clearing clearing solution [8g Sodium Dodecyl Sulfate, 1.25g Boric Acid, H2O /100ml, pH 8.4, filter with 2μm filter]. The samples were then washed in PBS overnight at room temperature and sequentially incubated with the following antibodies: anti-Troponin T (Sigma-Aldrich, T6277), Alexa Fluor anti-mouse IgG 488 (Invitrogen). Each antibody was incubated with samples for 2-3 days on a 37°C shaker at a dilution of 1:200 in 6% BSA in PBS, 0.2% Triton X-100, and 0.01% sodium azide. The samples were mounted with X-CLARITY Mounting Solution (Logos Biosystems, # C13101). Images were acquired by using a Zeiss LSM 780 microscope and Zen software (Carl Zeiss Microscopy).

Ultrastructural analysis

Adult fish hearts for ultrastructural analysis were properly dissected out and fixed in Trump's fixative solution (4% paraformaldehyde and 1% glutaraldehyde in 0.1 M phosphate buffer [pH 7.2]) at room temperature for 1 hour, followed by overnight incubation at 4°C. Fixed samples were subsequently processed and imaged at the Mayo Clinic Electron Microscopy Core Facility using a Philips JEOL 1400+ transmission electron microscope.(11)

MMEJ-based single guide RNA design and F0 injection

Sequences for genes of interest were acquired and downloaded from the Ensemble (http://useast.ensembl.org/index.html). Preferred exons were uploaded to an online algorithm, MENTHU (http://genesculpt.org/menthu/) for selecting guide RNA. sgRNA sequences are listed in Figure 4A and Table S2. Single guide RNAs (sgRNAs) with appropriate chemical modifications were synthesized and obtained from Synthego (Synthego Corporation). sgRNAs were dissolved in nuclease-free duplex buffer (Integrated DNA Technologies, 11-01-03-01). 100 μM sgRNAs were used as stock solutions, which were diluted to 5 μM or 10 μM as working solutions. The Alt-R Cas9 protein (Integrated DNA Technologies, 1081058) was diluted to 3.3 μg/μL in buffer (20 mM HEPES, 100 mM NaCl, 5 mM MgCl2, 0.1 mM EDTA at pH 7.5). Final concentration of 5 μM sgRNA and 300 ng Cas9 protein were then mixed and incubated in a 37°C water bath for 10 min to assemble the sgRNA-Cas9 protein (sgRNP) complex. ~3 nL of the sgRNP complex with 0.01% phenol red indicator was then injected into one-cell stage fish embryos to obtain F0 MMEJ injected embryos.

Knockout (KO) score calculation

Either individual embryos injected with an MMEJ sgRNP or tail fin from adult fish was collected for extracting genomic DNA (HotSHOT method).(12) 2 µL of resultant genomic DNA lysates were used as templates for PCR analysis to quantify KO score for each predicted MMEJ sgRNA. The PCR primer sequence information for KO score quantification is listed in Table S1. To quantify the score, 5 µL PCR product was digested with Exonuclease I (New England Biolabs,M0293) for enzymic purification and subsequently sent for Sanger sequencing at Genewiz (https://clims4.genewiz.com/CustomerHome/Index). The chromatograms from two PCR amplicons using either predicted MMEJ sgRNA injected or uninjected embryonic genomic DNA lysates as templates were analyzed for KO score calculation using the Inference of CRISPR Edits (ICE) v2 CRISPR Analysis Tool by TIDE (https://www.synthego.com/products/bioinformatics/crispr-analysis.(13) Percent microhomology allele was calculated by dividing the KO score for the predicted microhomology indel by the total KO score.

Generation of stable mutants

The F0-MMEJ injected mutant fish were raised to adulthood and outcrossed. The individual F1 adults were tested individually via sequencing the targeted genomic loci. Selected F1 containing the desirable mutations were outcrossed for 1-3 generations, aiming to eliminate potential off-target effects. A high-percentage (4%-6%) agarose gel-based method is then used for genotyping and detecting the small

CRISPR-Cas9 induced indels.(14) After loading the samples into each lane, power supply was set to 100 V, running time should be 2 hours. Gel images were acquired using a gel-documentation system (Analytik Jena US UVP UVsolo Touch).

SDS agarose gel electrophoresis

Protein samples from the single zebrafish larvae were extracted by homogenization using a mortar and pestle (Thermo Fisher Scientific) on the surface of dry ice. Protein lysates are then moved to 30 µl sample buffer [8 M urea, 3% SDS, 2 M Thiourea, 0.05 M Tris-HCl (pH 8.0), 0.03% Bromophenol Blue, 75 mM DTT and 0.01% protease inhibitors cocktail (Roche)]. After the samples were incubated at 65°C for 15 min and then be placed on ice for 2 mins. 0.5 µl Benzonase enzyme (Sigma Chemical Company #E1014-5KU) was added to digest the genomic DNA and to reduce viscosity, and then centrifuged at 4°C 12000 rpm for 10 min. The supernatants were separated on a 1.2% agarose gel [0.6g SeaKem Gold Agarose (Lonza #50512) heated in 10 mL 5x TGS buffer (0.25M Tris, 1.92M glycine, 2M SDS) and 27 mL dH_2O, mixed with 15 mL 99.5% glycerol and keep at 65℃ till use] using a vertical agarose gel electrophoresis system;(15) 1 kb Plus DNA Ladder (Invitrogen) was used as size markers. The gel was fixed in a prefixing solution [50% methanol, 12% glacial acetic acid, 5% glycerol] for 20 mins, washed with water for 30 mins, dried in an oven at 50°C overnight, transferred to fixing solution [50% methanol, 10% glacial acetic acid, 10% Fixative Enhancer Concentrate (Bio-Rad, #1610461)], and visualized by a Silver Stain Plus Kit (Bio-Rad, #1610449).

Coomassie blue staining

A 2 dpf larvae were transferred to RIPA buffer (Sigma-Aldrich) and homogenized using a Bullet Blender tissue homogenizer (Next Advance). The resulting protein lysates were resolved on a mini-protein TGX precast gel, followed by Commassie blue staining using a standard protocol.(11)

Western blotting

Hearts dissected from adult fish were transferred immediately to RIPA buffer (Sigma-Aldrich) supplemented with protease inhibitor cocktail (Roche), 1 mM PMSF (Thermo Fisher, # 36978), and homogenized using 10-15 stainless steel beads (0.5 mm diameter, SSB05, Next Advance) in a Bullet Blender tissue homogenizer (Next Advance), speed 8 for 4 minutes and then speed 10 for 2 minutes. The protein lysates were subjected to western blotting using a standard protocol.(9) Samples were separated by sodium dodecyl sulfate-polyacrylamide gel electrophoresis (SDS-PAGE) and then transferred onto a PVDF membrane (Millipore, CA, USA). The following primary antibodies were used: anti-LC3-II (1:3000, Cell Signaling Technology, 12741s) , ubiquitin(1:1000, Invitrogen, #PA5-17067), anti-Atg7(1:1000, Sigma, A2856), anti-Ulk1(1:1000, Cell Signaling Technology, 8054T), anti-β-actin −Peroxidase antibody (1:1000, Sigma, A3854), anti-GAPDH (HRP Conjugate) (1:1000, Cell Signaling Technology, 3683s), anti-Drp1 (1:1000, Cell Signaling Technology, 8750s), anti-Tom20 (1:1000, Cell Signaling Technology, 42406T), mouse anti-rabbit IgG-HRP (1:1000, Santa Cruz Biotechnology, sc-2357).

Measurements of cardiomyocyte size and nuclei size

Fluorescent immunostaining using an anti-β-Catenin (1:200, Sigma, C7207) for the cell membrane and an anti-MEF2(A+C) (1:200, Abcam, ab197070) for cardiomyocyte (CM) nuclei was performed in isolated embryonic zebrafish hearts according to a protocol described previously to measure the CM size and nuclei.(3, 16) A Zeiss Axioplan 2 microscope was used for imaging. 15 cells in the outer curvature region (OCR) of each individual heart were selected for measuring the cell surface area using ImageJ software.(3, 17) Only CMs with clear outlines were chosen for the measurement. Measurements from three fishes per group were obtained to calculate the average cardiomyocyte size and nuclei size.

Supplemental tables and figures

Table S1. Genotyping primers.

Gene	Primer Name	Sequence
ttna-null	ttna-N1F	5'-CGCACCAGTTGTTACTGTC-3'
	ttna-C2R	5'-CATAGTCAGTCTGAACACAAGG-3'
ttnb-null	ttnb-N2F	5'-CAGCAAAAATCACTTTATTCTG-3'
	ttnb-cp-R2	5'-CAAAATGGTGCAGAACTTATGG-3'
ttnd-null	ttna-N1F	5'-CGCACCAGTTGTTACTGTC-3’
	ttnb-cp-R2	5'-CAAAATGGTGCAGAACTTATGG-3’
ulk1a	ulk1a^{e6}-F	5'-CTGATCCATAACCAATCTGCG-3'
	ulk1a^{e6}-R	5'-GTCTGAGTGAGGACACCATC-3'
atg7	atg7^{e6}-F	5’-GATTGCGTTTCATGTGTCG-3’
	atg7^{e6}-R	5’-TCTGCCACAAATGTTACTGATG-3’
anf	Qnppa-F	5'-GATGTACAAGCGCACACGTT-3'
	Qnppa-R	5'-TCTGATGCCTCTTCTGTTGC-3'
bnp	Qnppb-F	5'-CATGGGTGTTTTAAAGTTTCTCC-3'
	Qnppb-R	5'-CTTCAATATTTGCCGCCTTTAC-3'
vmhc	Qvmhc-F	5'-TCAGATGGCAGAGTTTGGAG-3'
	Qvmhc-R	5'-GCTTCCTTTACAGTTACAGTCTTTC-3'
vmhcl	QvmhcL-F	5'-GCGATGCTGAAATGTCTGTT-3'
	QvmhcL-R	5'-CAGTCACAGTCTTGCCTCCT-3'
ulk1a	Qulk1a^{e6}-F	5'-CACCATCCGTGTGTTACTGC-3'
	Qulk1a^{e6}-R	5'-TAGTGTGGCAGCCATTGTGT-3'
	Qulk1a^{e25}-F	5'-GCTGGCATCAAAGAAGGAAA-3'
	Qulk1a^{e25}-R	5'-CTGCCGTCTGTACCATCTGA-3'

Table S2. Supporting data related to the F0-based MMEJ screen

Target genes	Design Exon	sgRNA sequence	Frame Shift	Deletion sequence	KO score (%)
atg7	Exon 6	CTGTGCCTCCAGCGGAACGACGG	Yes	AACGACGG	86
ulk1a	Exon 6	ATCCTTCTCTCATACAGCACAGG	Yes	CACAG	92

Supplemental movie. Cardiac phenotypes in homozygous *ttntv* embryos

Shown are differential interference contrast movies of WT and *ttntv* embryos. Shown in the first two rows are 2 dpf larvae and shown in the bottom row are 9 dpf larvae. 60 frames per second.

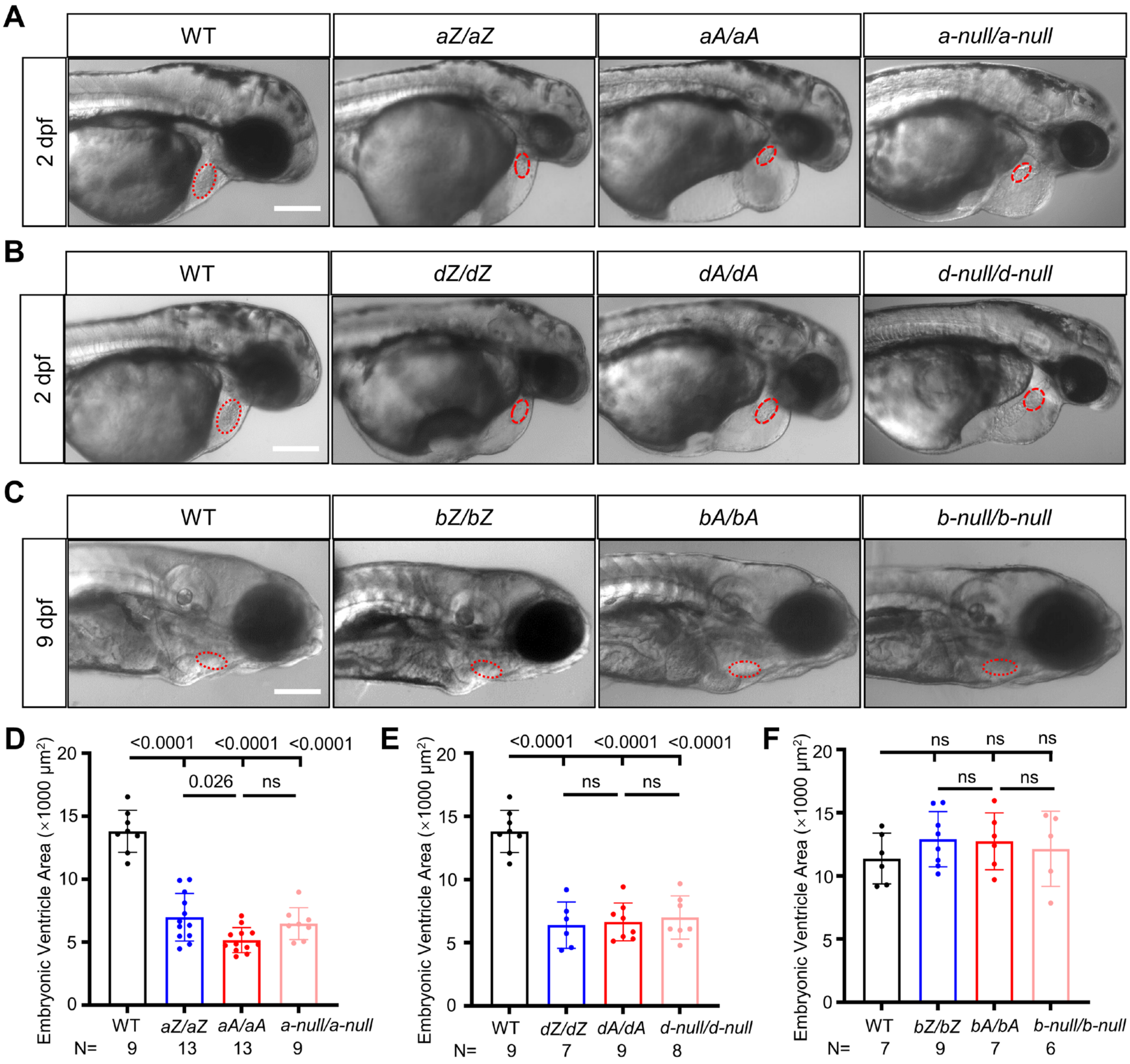

Figure S1. Pericardiac edema, somite deformation and abnormal ventricle size are detected in *ttntv-a* and *ttntv-d*, but not in *ttntv-b*.

(A-B) Differential interference contrast images of 2 dpf embryos show that the pericardium edema is the common phenotype in all the *TTNtv* mutations but not in WT. Pericardium edema and yolk edema do not belong to a specific mutation. Ventricle size become small, and the heart shape become abnormal in mutation groups, but no difference in each mutation group. (C) Differential interference contrast images of 9 dpf embryos show no pericardium edema, yolk edema, ventricle size, or ventricle shape difference in WT and mutation groups. Scale bar, 200 μm. (D) Quantification of the ventricle area in Figure S1-A. (E) Quantification of the ventricle area in Figure S1-B.(F) Quantification of the ventricle area in Figure S1-C. One-way ANOVA was used to compare multiple groups for each mutation. Data are presented as the mean ± SD. WT, wild type. ns, non-significant.

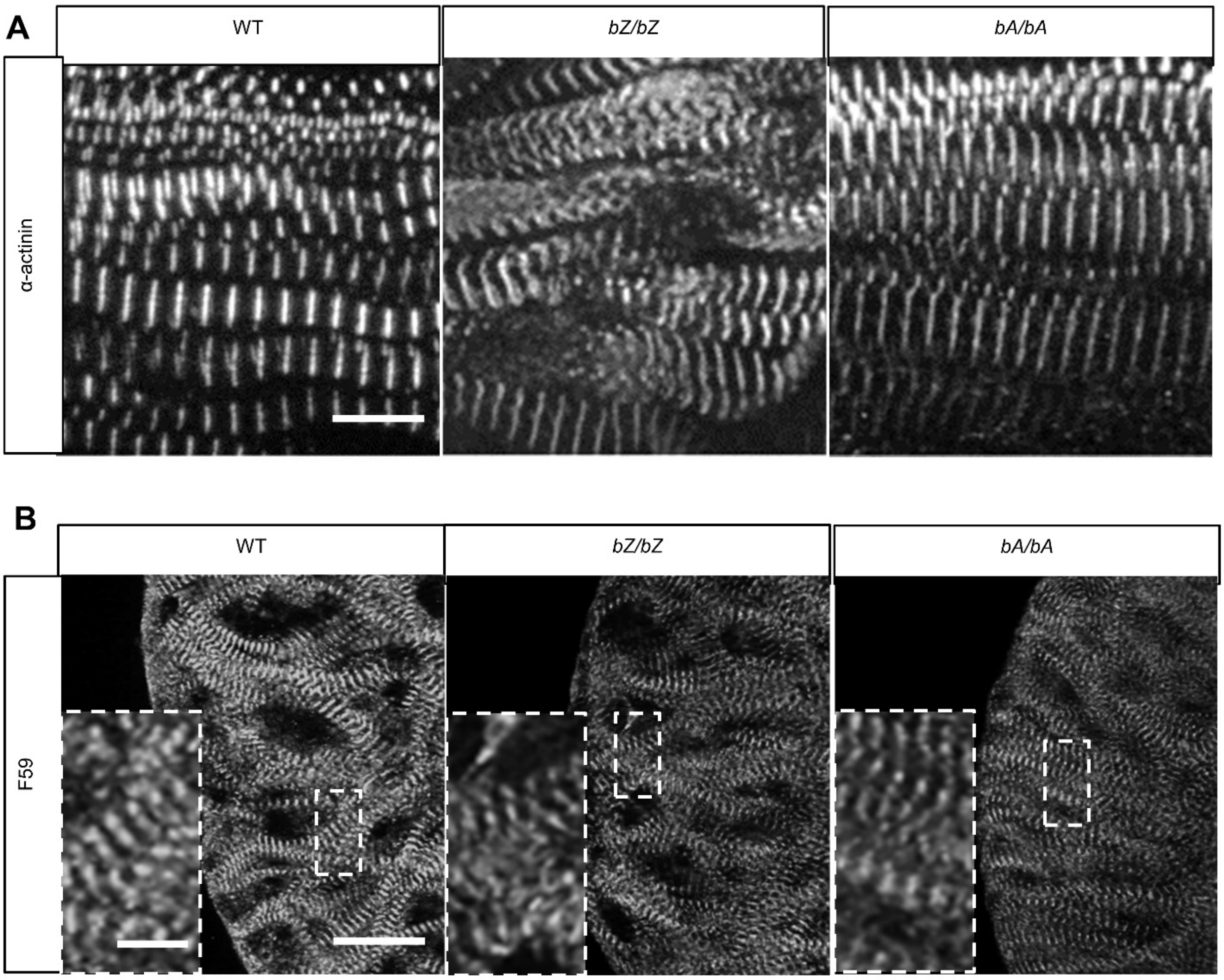

Figure S2. Sarcomere phenotypes in homozygous *bZ* and *bA*

(A) Shown are α-actinin antibody immunostaining of the embryonic somites. Similar to our previous data,(18) *bZ/bZ* but not *bA/bA* and WT exhibits disrupted sarcomere in somites at 9 dpf. Scale bar= 20 μm. (B) Shown are F59 antibody immunostaining of the 9 dpf embryonic heart ventricle. *bZ/bZ* and *bA/bA* do not show significant difference on sarcomere structure with WT. Scale bar=2 μm. Insets are enlarged images of the encircled region. Scale bar=0.5 μm.

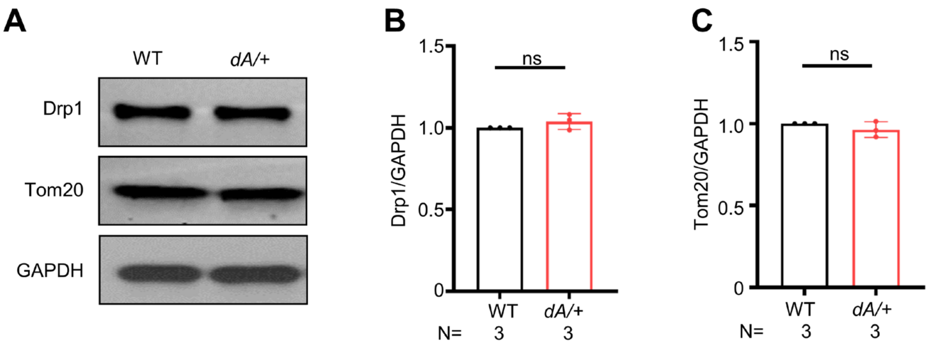

Figure S3. Mitochondria dysfunction was not detected at an early stage of *ttntv-A* DCM model.

(A-C) Representative western blot and quantification of Drp1 and Tom20 from zebrafish hearts at 3 months. Independent samples T-test is applied to compare WT and *dA/+*. Data are presented as mean ± SD. WT, wild type. ns, non-significant.

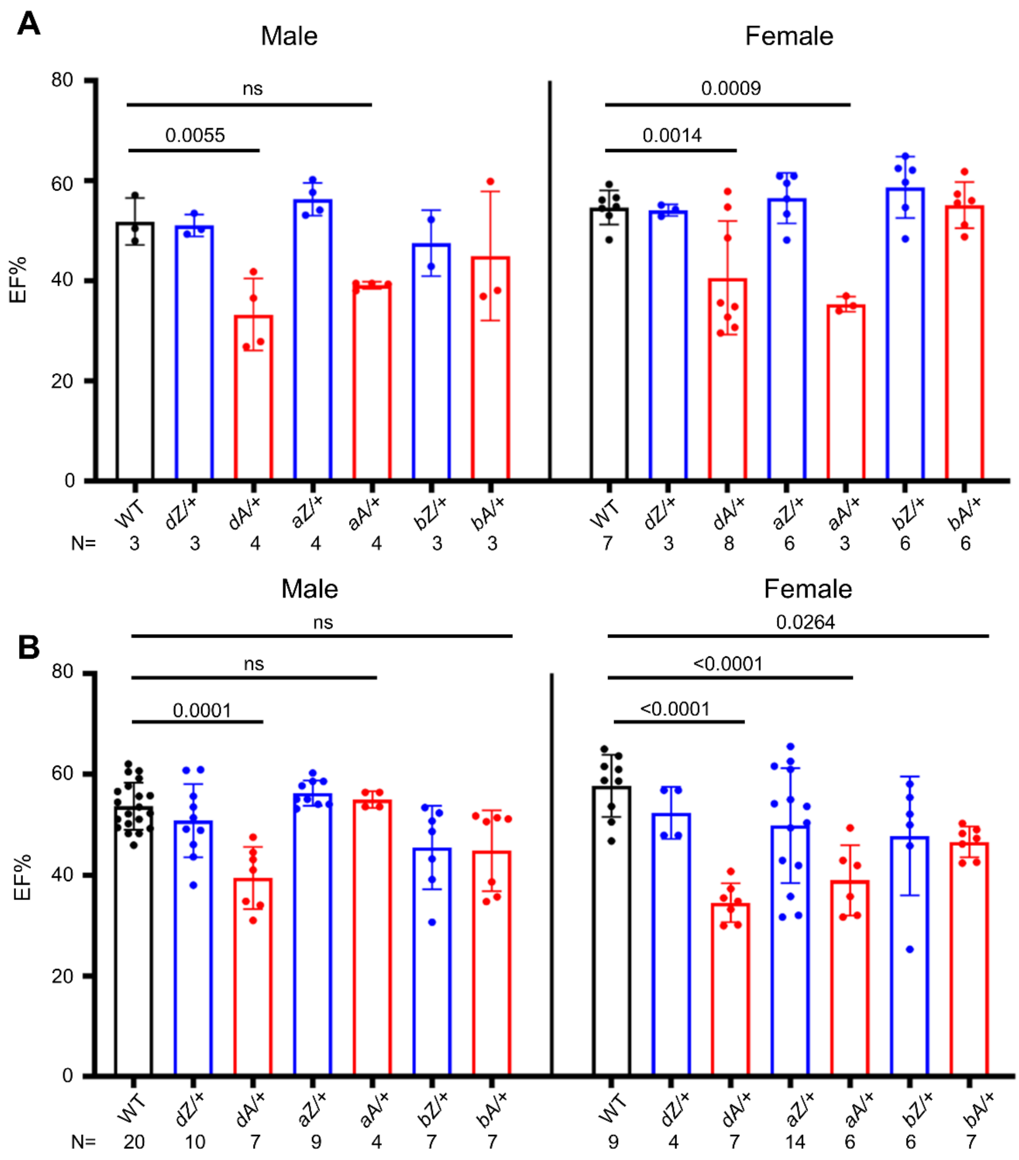

Figure S4. AH in cardiac dysfunction is more frequently noted in female than male fish

(A) High-frequency echocardiography was performed in fish at 3-month of age. Female *dA/*+ and *aA/*+ fish manifest severer cardiac dysfunction than male fish. (B) High-frequency echocardiography was performed at 6-month of age. Female *dA/*+*,* *aA/*+ and *bA/*+ fish show more markedly reduction of EF than male fish. One-way ANOVA was used to compare multiple groups for each mutation. Data are presented as mean ± SD. WT, wild type. ns, non-significant.

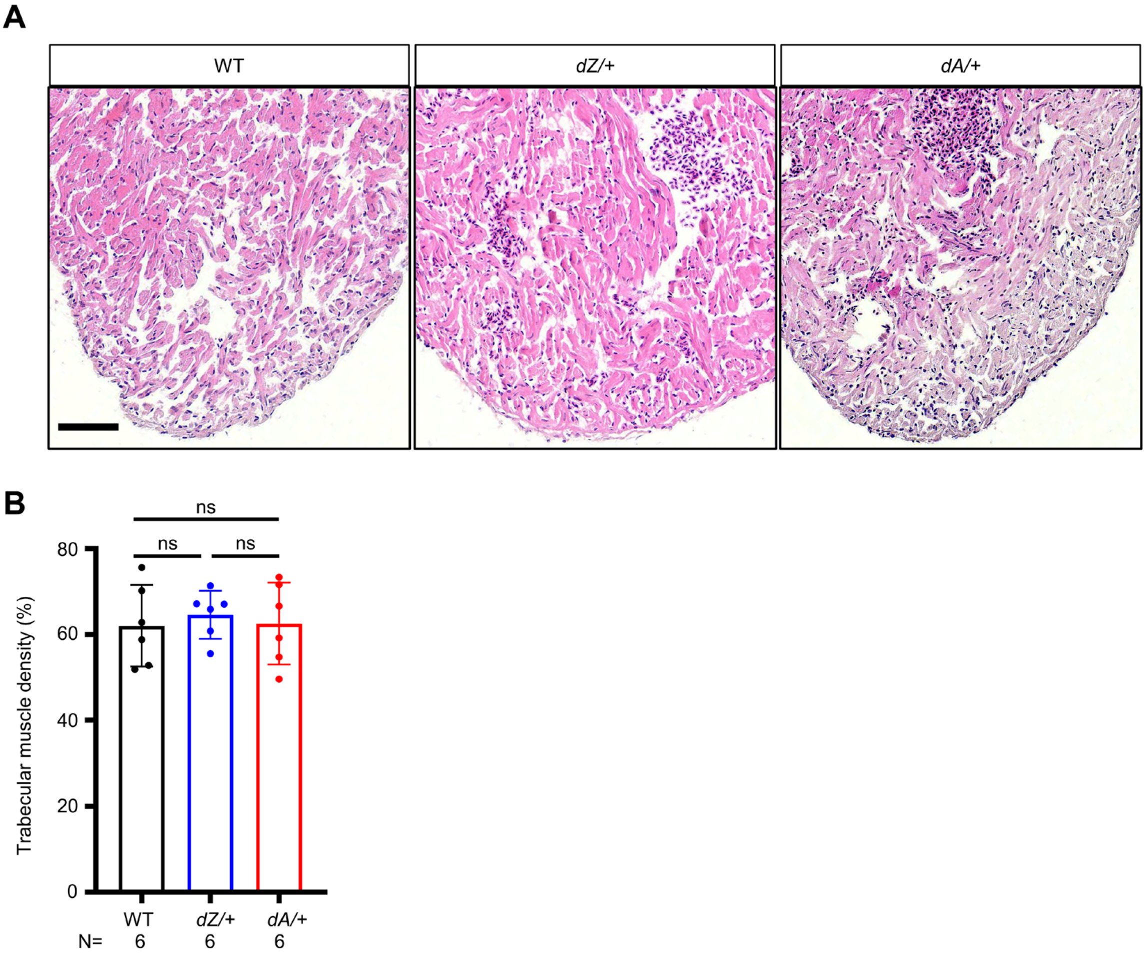

Figure S5. Trabecular muscle density was unchanged in the ttntv heterozygous fish.

(A-B) Representative images of H&E staining in the apex area of ventricle and quantification of trabecular muscle density in fish at 3 months. Scale bar =50 µm. One-way ANOVA was used to compare multiple groups for each mutation. Data are presented as the mean ± SD. WT, wild type. ns, non-significant.

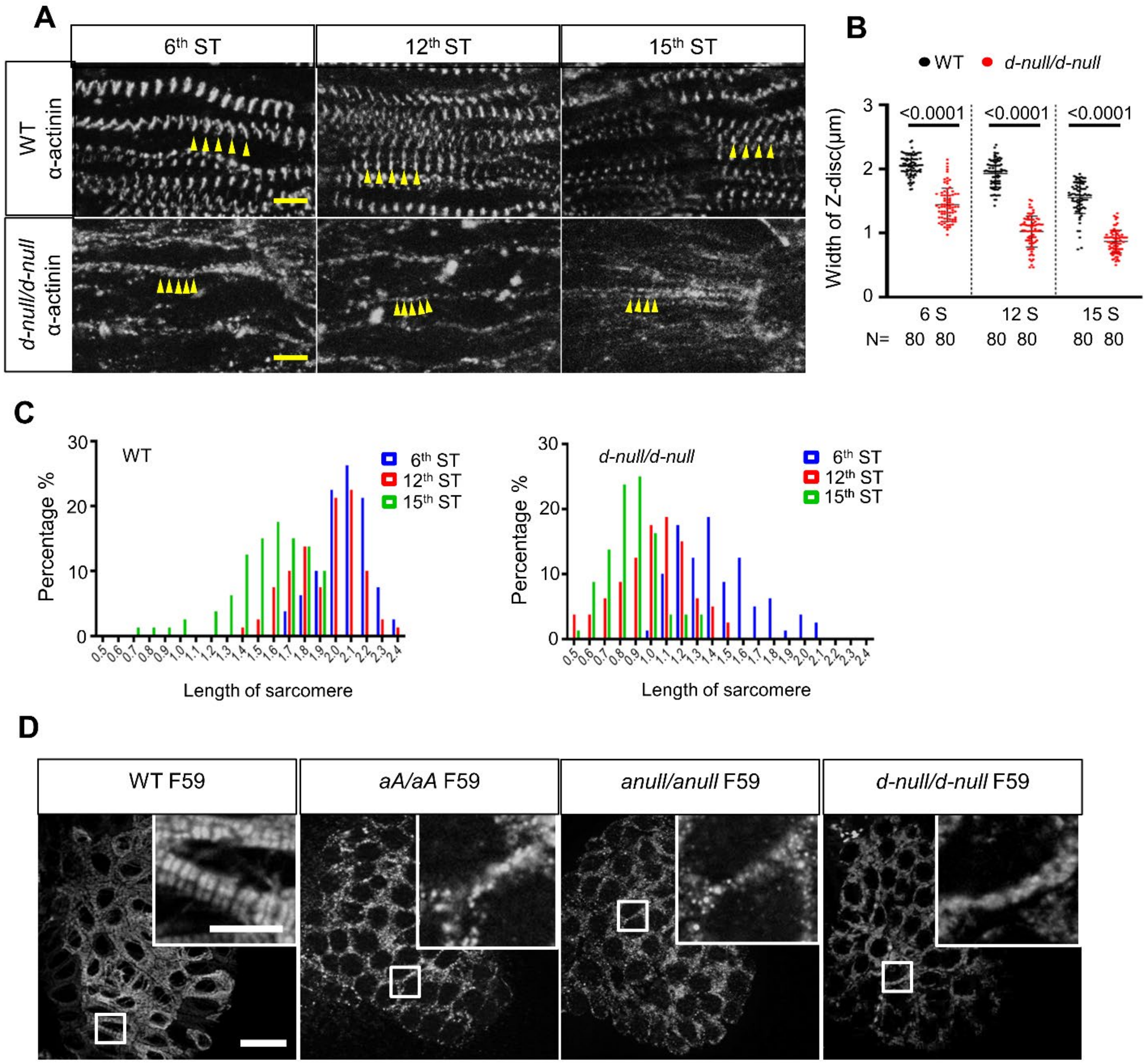

Figure S6. *d-null/d-null* manifests sarcomeric defects

(A) Shown are anti-α-actinin immunostaining of WT and *d-null/d-null* mutant embryos at the 18-somite (ST) stage. Images were captured at the 6th, 12th and 15th somites, respectively, representing three different stages of myofibrillogenesis. Yellow arrowheads indicate the striated sarcomere. Scale bar= 5 μm. (B) Quantification of width of the Z-discs of WT and *d-null/d-null* mutant at the 6th, 12th and 15th somites. Independent samples T-test is applied to compare the difference between WT and *d-null/d-null*. Data are presented as mean ± SD. WT, wild type. (C) Frequency distributions of length of sarcomere in 6th, 12th, and 15th somites of WT and *d-null/d-null,* as measured by the distance between neighboring two yellow arrowheads in panel A. n=80 for each group in different somites. (D) Comparison of cardiac sarcomere structure in 2 dpf embryos via F59 antibody staining. Scale bar=10 μm. Insets show enlarged images of the area circled with dashed lines. Scale bar=5 μm.

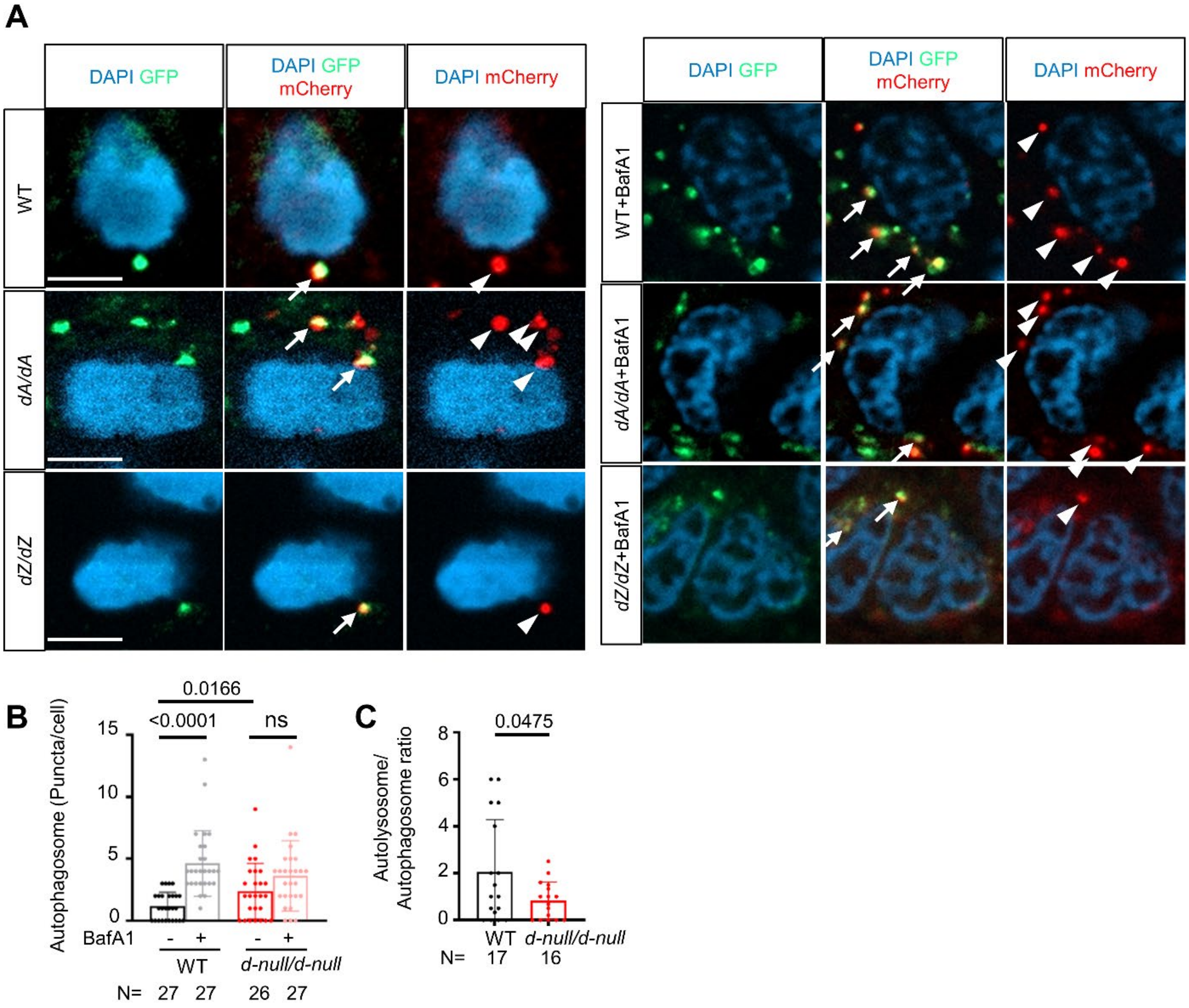

Figure S7. Additional data for Figure 5.

(A) Representative images of a cardiomyocyte in a embryonic heart injected with a *mCherry-EGFP-LC3II* tandem plasmid. The arrow indicates a yellow puncta with both GFP and mCherry that presumably represents an autophagosome, and the arrowhead indicates a red puncta with mCherry that presumably represents an autolysosome. Scale bar: 5 µm. (B) Autophagy regulation in *d-null/d-null* embryos. Quantification of the autophagosome puncta in 2-dpf *d-null/d-null* embryos with BafA1 treatment. One-way ANOVA was used to compare multiple groups. (C) The autolysosome/autophagosome ratio in 2-dpf *d-null/d-null* embryos without BafA1-treatment. Independent samples T-test is applied. Data are presented as mean ± SD. WT, wild type. ns, non-significant.

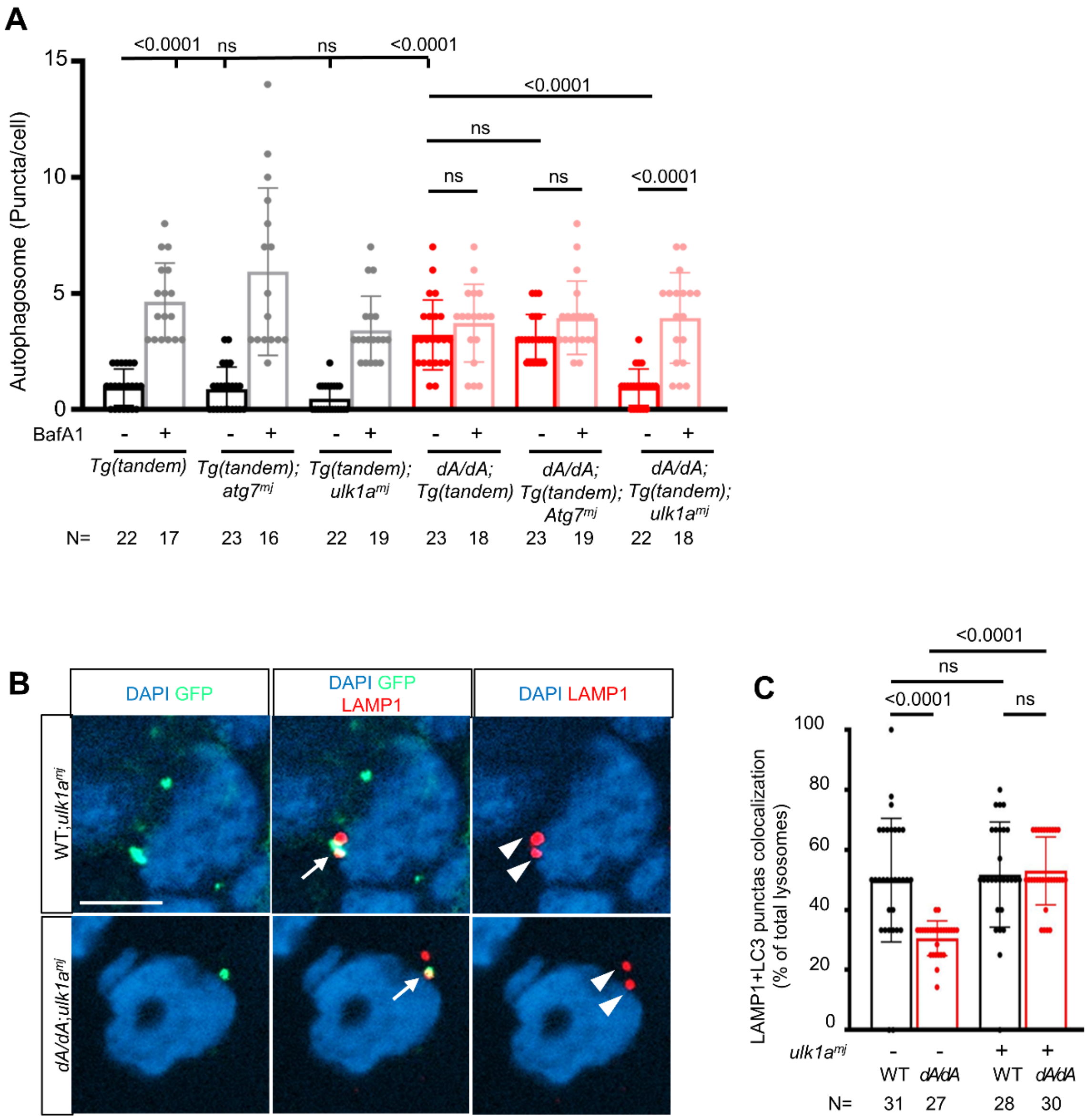

Figure S8. An F0-based MMEJ assessment of candidate genes related to autophagy regulation in *ttntv-A* and the fusion-rescuring effects of *ulk1a*.

(A) Quantification of autophagosome puncta in zebrafish embryos co-injected with *mCherry-EGFP-LC3II* tandem plasmid and sgRNAs against autophagy genes. *ulk1a*^{mj} but not *atg7*^{mj} rescued autophagy dysregulation by reducing the basal level of autophagy and increasing autophagic flux. (B) Representative image and quantification of LC3-GFP and lysosome marker LAMP1 colocalization compared with the total lysosome. The arrow indicates a yellow puncta with both GFP and LAMP1 that represents co-localization, and the arrowhead indicates a red puncta with only LAMP1. Scale bar: 5 μm. (C) Quantification of (B), depletion of *ulk1a* rescues the impairment of autophagosome-lysosome fusion in *dA/dA* hearts. One-way ANOVA was used to compare multiple groups. Data are presented as the mean ± SD. WT, wild type. ns, non-significant.

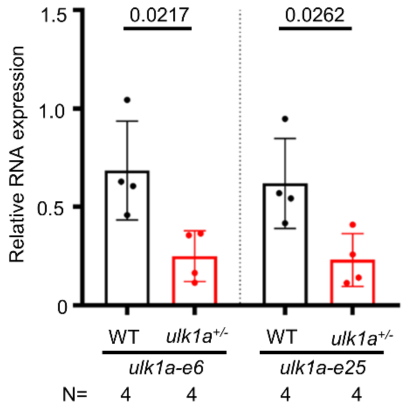

Figure S9. Expression levels of *ulk1a* transcripts

Transcripts of *ulk1a* are reduced in *ulk1a*$^{+/-}$. The expression levels of *ulk1a* transcripts were measured by quantitative RT–PCR. Primer pairs are located in exon 6 and exon 25 of *ulk1a*. Independent samples T-test is applied to compare the difference between WT and *ulk1a*$^{+/-}$. Data are presented as mean ± SD. WT, wild type.

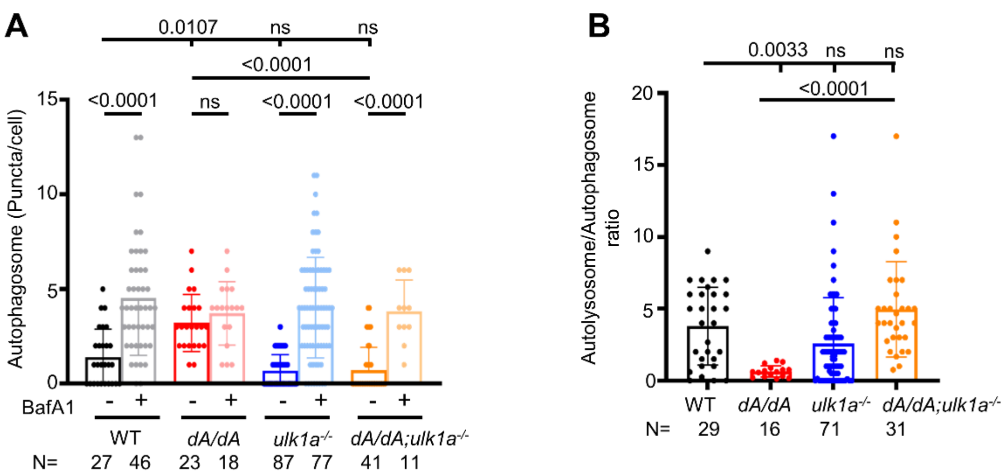

Figure S10. *ulk1a* homozygous mutant repairs autophagy dysregulation in embryonic stage *ttntv* mutants

(A) Quantification of the autophagosome puncta in 2-dpf embryos either with or without BafA1 treatment. (B) The autolysosome/autophagosome ratio of BafA1 untreated groups. *ulk1a*$^{-/-}$ can reduce the basal autophagosome level of *dA/dA* and can recover the autophagic flux affected by *dA/dA*. One-way ANOVA was used to compare multiple groups for each mutation. Data are presented as mean ± SD. WT, wild type. ns, non-significant.

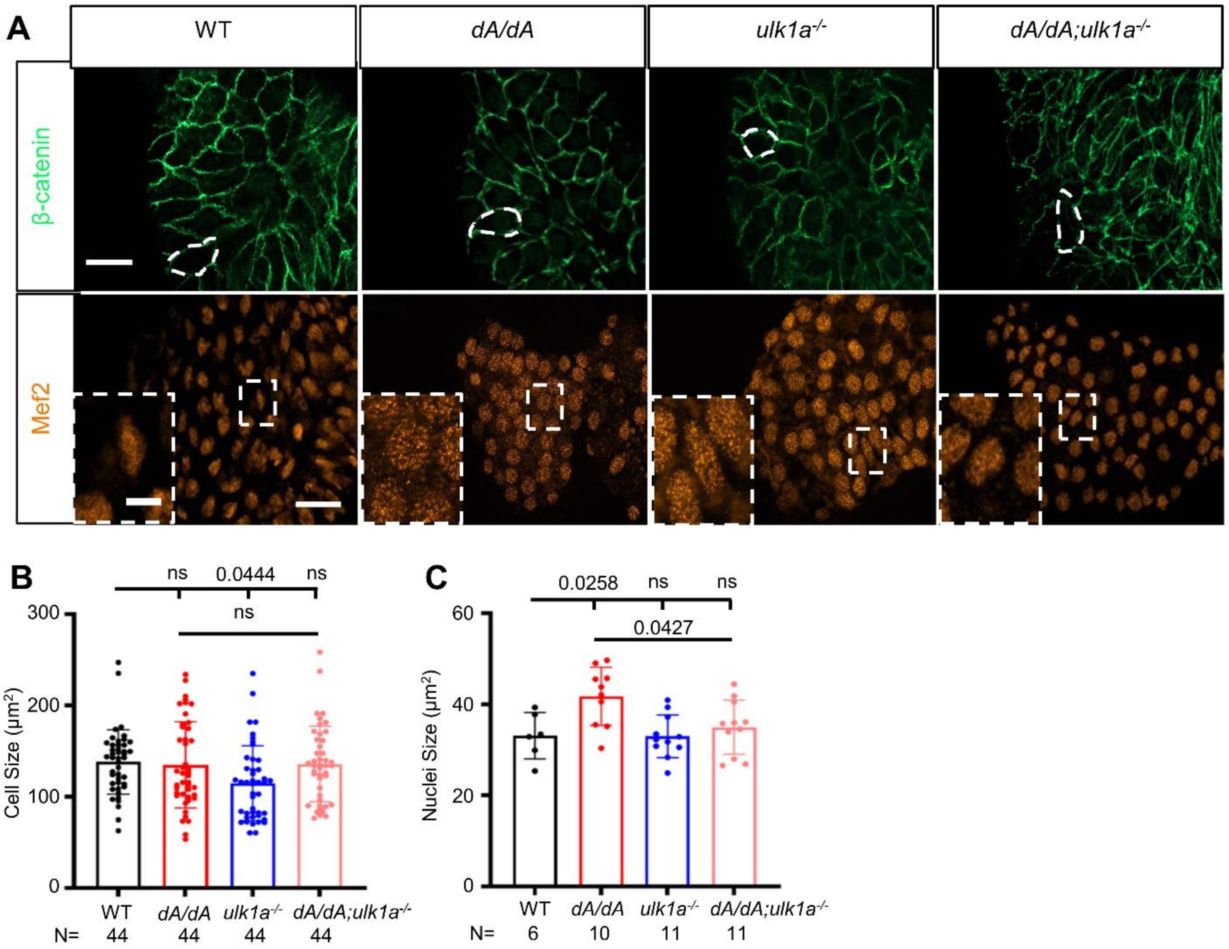

Figure S11. *ulk1a* mutant attenuates the enlarged nuclear size in *dA/dA* ventricles

(A) Representative images of hearts from 2-dpf embryos after immunostaining. The nucleus and membrane of cardiomyocytes (CMs) were identified by immunostaining with anti-myocyte enhancer factor-2 (Mef2) (orange) and anti-β-catenin (green) antibodies, respectively. Representative CMs in the outer curvature are outlined by the dashed white lines. Insets are higher magnification images of the boxed areas. Scale bar =10 μm. Scale bar in insets is 2.5 μm. (B) Quantification of the CM cell area based on anti-β-catenin staining. (C) Quantification of the nuclear area based on anti-Mef2 staining. One-way ANOVA was used to compare multiple groups for each mutation. Data are presented as the mean ± SD. WT, wild type. ns, non-significant.

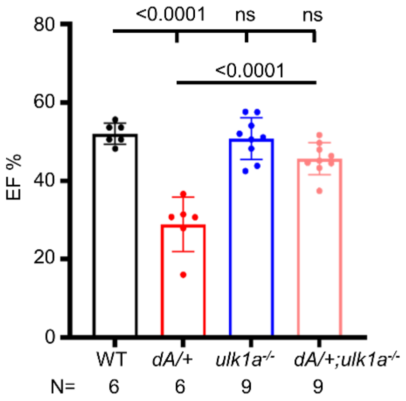

Figure S12. *ulk1a* homozygous mutant repairs cardiac dysfunction in *ttntv-A* adult fish.

High-frequency echocardiography was performed on 3-month-old zebrafish to quantify EF%. One-way ANOVA was used to compare multiple groups for each mutation. Data are presented as mean ± SD. WT, wild type. ns, non-significant.

Supplemental references

1. Asquith B, and Bangham CR. An introduction to lymphocyte and viral dynamics: the power and limitations of mathematical analysis. *Proc Biol Sci.* 2003;270(1525):1651-7.
2. Hoage T, Ding Y, and Xu X. Quantifying cardiac functions in embryonic and adult zebrafish. *Methods Mol Biol.* 2012;843:11-20.
3. Yang JC, and Xu XL. Immunostaining of Dissected Zebrafish Embryonic Heart. *Jove-J Vis Exp.* 2012(59).
4. Wang LW, Huttner IG, Santiago CF, Kesteven SH, Yu ZY, Feneley MP, et al. Standardized echocardiographic assessment of cardiac function in normal adult zebrafish and heart disease models. *Dis Model Mech.* 2017;10(1):63-76.
5. Zhang H, Dvornikov AV, Huttner IG, Ma X, Santiago CF, Fatkin D, et al. A Langendorff-like system to quantify cardiac pump function in adult zebrafish. *Dis Model Mech.* 2018;11(9).
6. Ma X, and Xu X. A Swimming-based Assay to Determine the Exercise Capacity of Adult Zebrafish Cardiomyopathy Models. *Bio Protoc.* 2021;11(15):e4114.
7. Sun YY, Fang YH, Xu XL, Lu GP, and Chen ZY. Evidence of an Association between Age-Related Functional Modifications and Pathophysiological Changes in Zebrafish Heart. *Gerontology.* 2015;61(5):435-47.
8. Wang JH, Panakova D, Kikuchi K, Holdway JE, Gemberling M, Burris JS, et al. The regenerative capacity of zebrafish reverses cardiac failure caused by genetic cardiomyocyte depletion. *Development.* 2011;138(16):3421-30.

9. Ding Y, Dvornikov AV, Ma X, Zhang H, Wang Y, Lowerison M, et al. Haploinsufficiency of mechanistic target of rapamycin ameliorates bag3 cardiomyopathy in adult zebrafish. *Dis Model Mech.* 2019;12(10).

10. Chung K, Wallace J, Kim SY, Kalyanasundaram S, Andalman AS, Davidson TJ, et al. Structural and molecular interrogation of intact biological systems. *Nature.* 2013;497(7449):332-+.

11. Bu H, Ding Y, Li J, Zhu P, Shih YH, Wang M, et al. Inhibition of mTOR or MAPK ameliorates vmhcl/myh7 cardiomyopathy in zebrafish. *JCI Insight.* 2021;6(24).

12. Truett GE, Heeger P, Mynatt RL, Truett AA, Walker JA, and Warman ML. Preparation of PCR-quality mouse genomic DNA with hot sodium hydroxide and tris (HotSHOT). *Biotechniques.* 2000;29(1):52, 4.

13. Brinkman EK, and van Steensel B. Rapid Quantitative Evaluation of CRISPR Genome Editing by TIDE and TIDER. *Methods Mol Biol.* 2019;1961:29-44.

14. Bhattacharya D, and Van Meir EG. A simple genotyping method to detect small CRISPR-Cas9 induced indels by agarose gel electrophoresis. *Sci Rep.* 2019;9(1):4437.

15. Warren CM, Krzesinski PR, and Greaser ML. Vertical agarose gel electrophoresis and electroblotting of high-molecular-weight proteins. *Electrophoresis.* 2003;24(11):1695-702.

16. Yang JC, Hartjes KA, Nelson TJ, and Xu XL. Cessation of contraction induces cardiomyocyte remodeling during zebrafish cardiogenesis. *Am J Physiol-Heart C.* 2014;306(3):H382-H95.

17. Auman HJ, Coleman H, Riley HE, Olale F, Tsai HJ, and Yelon D. Functional modulation of cardiac form through regionally confined cell shape changes. *Plos Biol.* 2007;5(3):604-15.

18. Shih YH, Dvornikov AV, Zhu P, Ma X, Kim M, Ding Y, et al. Exon- and contraction-dependent functions of titin in sarcomere assembly. *Development.* 2016;143(24):4713-22.
