# Supplementary material for: Allelic heterogeneity of TTNtv dilated cardiomyopathy can be modeled in adult zebrafish
Source: JCI Insight. 2024 Feb 27;9(7):e175501. doi: 10.1172/jci.insight.175501 (PMC11128207; doi:10.1172/jci.insight.175501)

Unedited gel pictures

Full unedited gel for Figure 4A

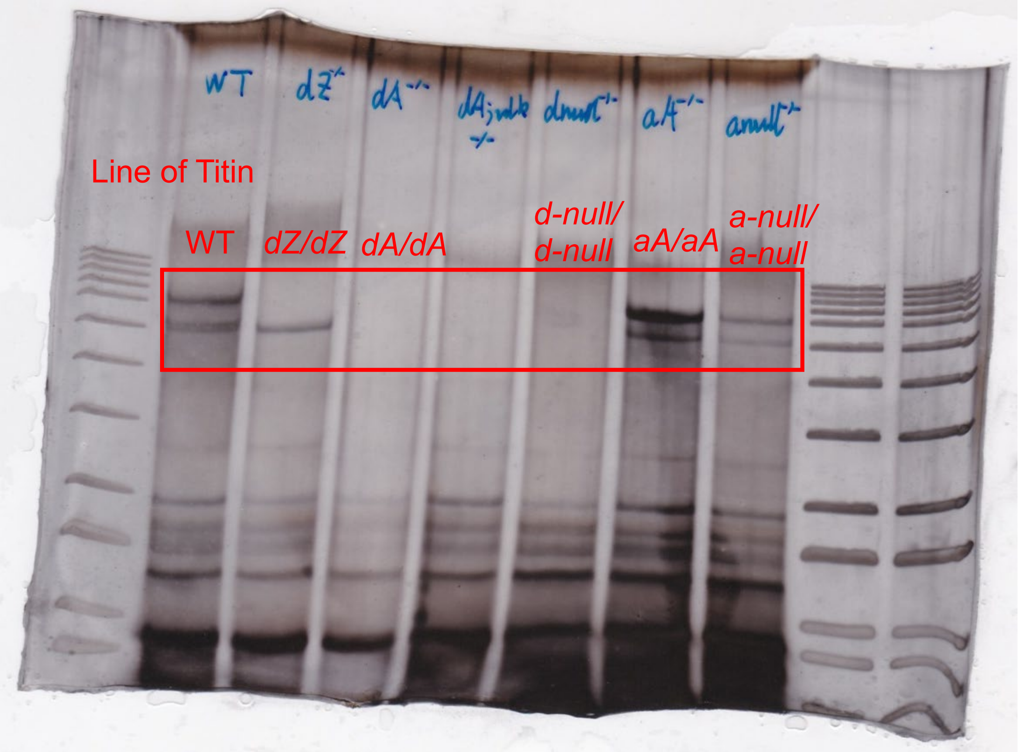

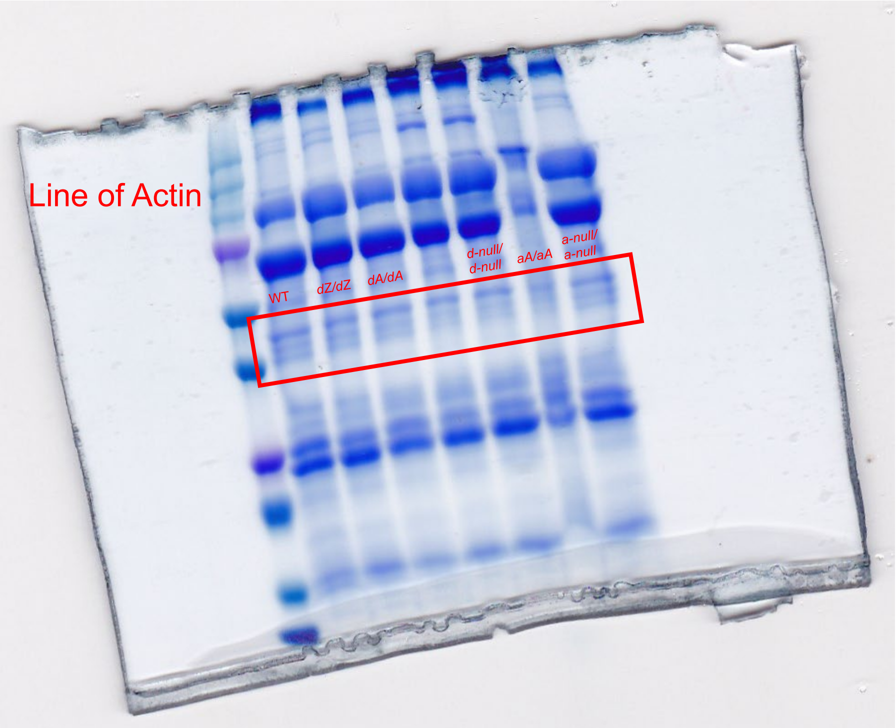

Full unedited gel for Figure 5A

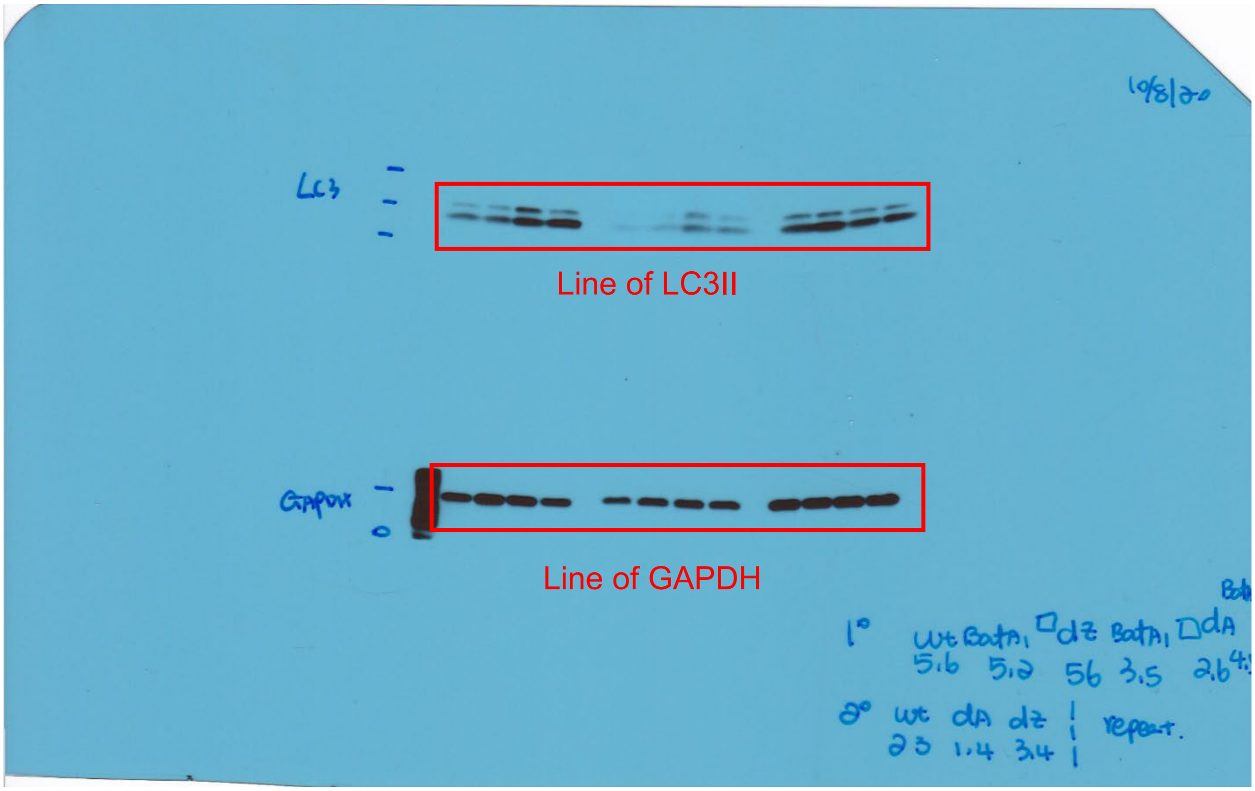

Full unedited gel for Figure 5E

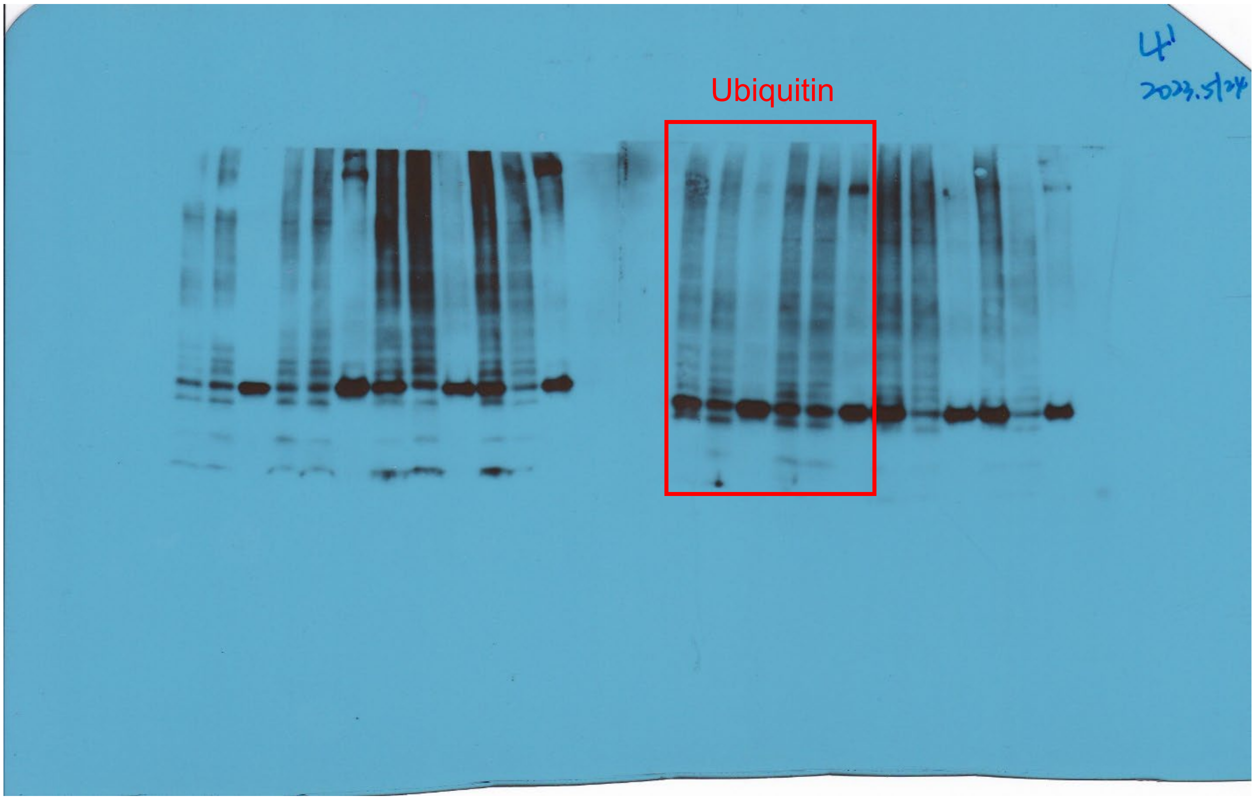

Full unedited gel for Figure 6D: atg7

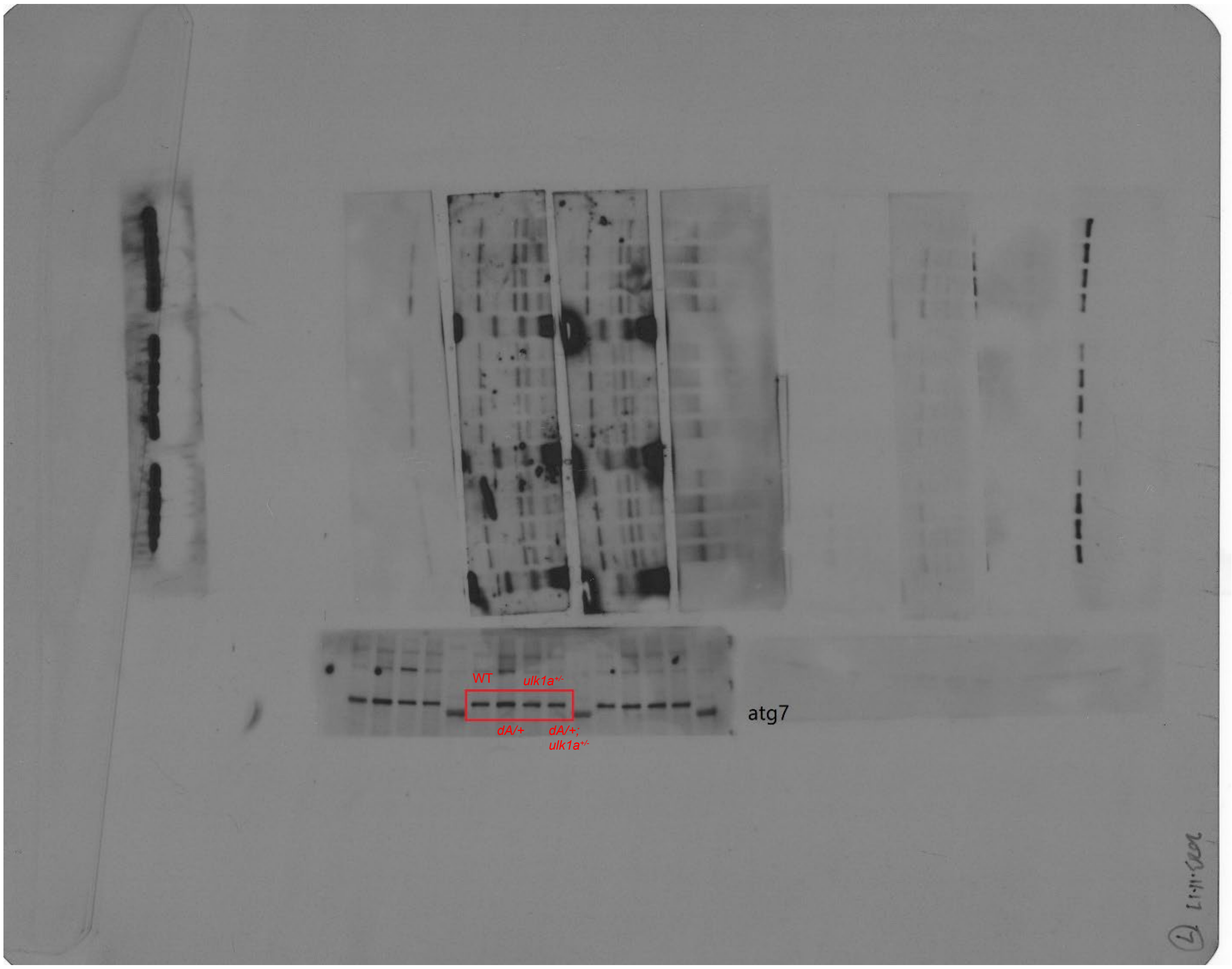

Full unedited gel for Figure 6D: ulk1

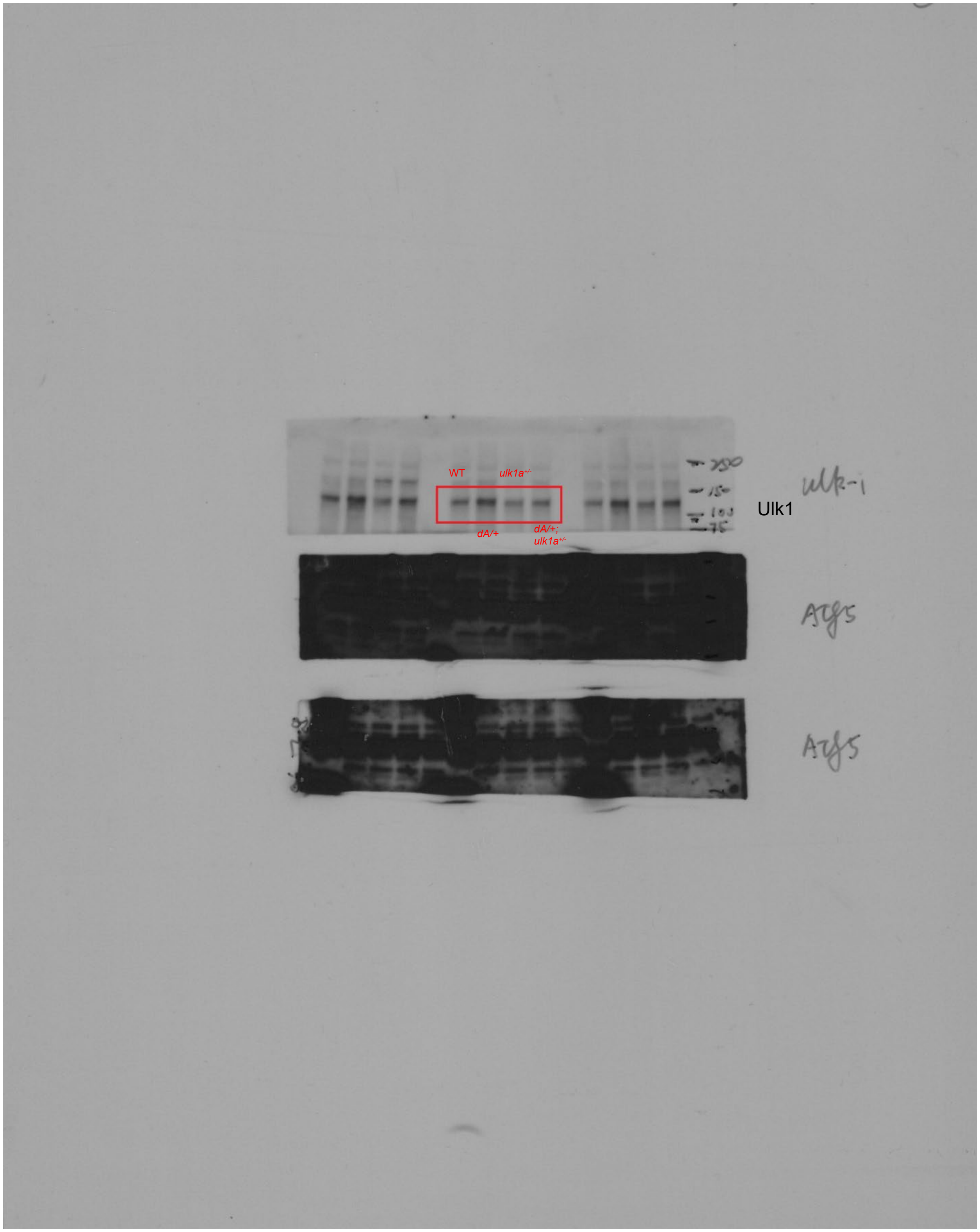

Full unedited gel for Figure 6D: GAPDH

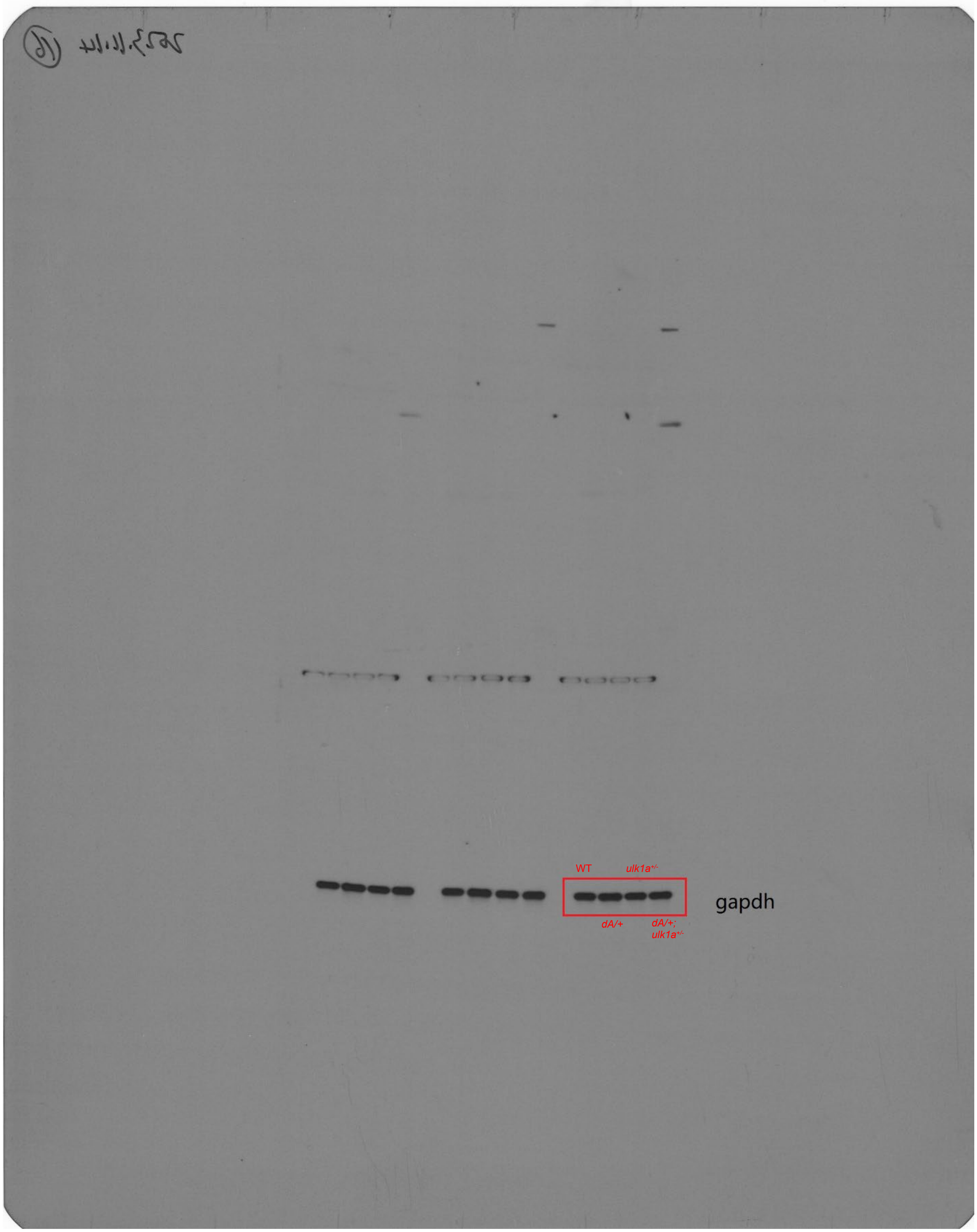

Full unedited gel for Figure 6G

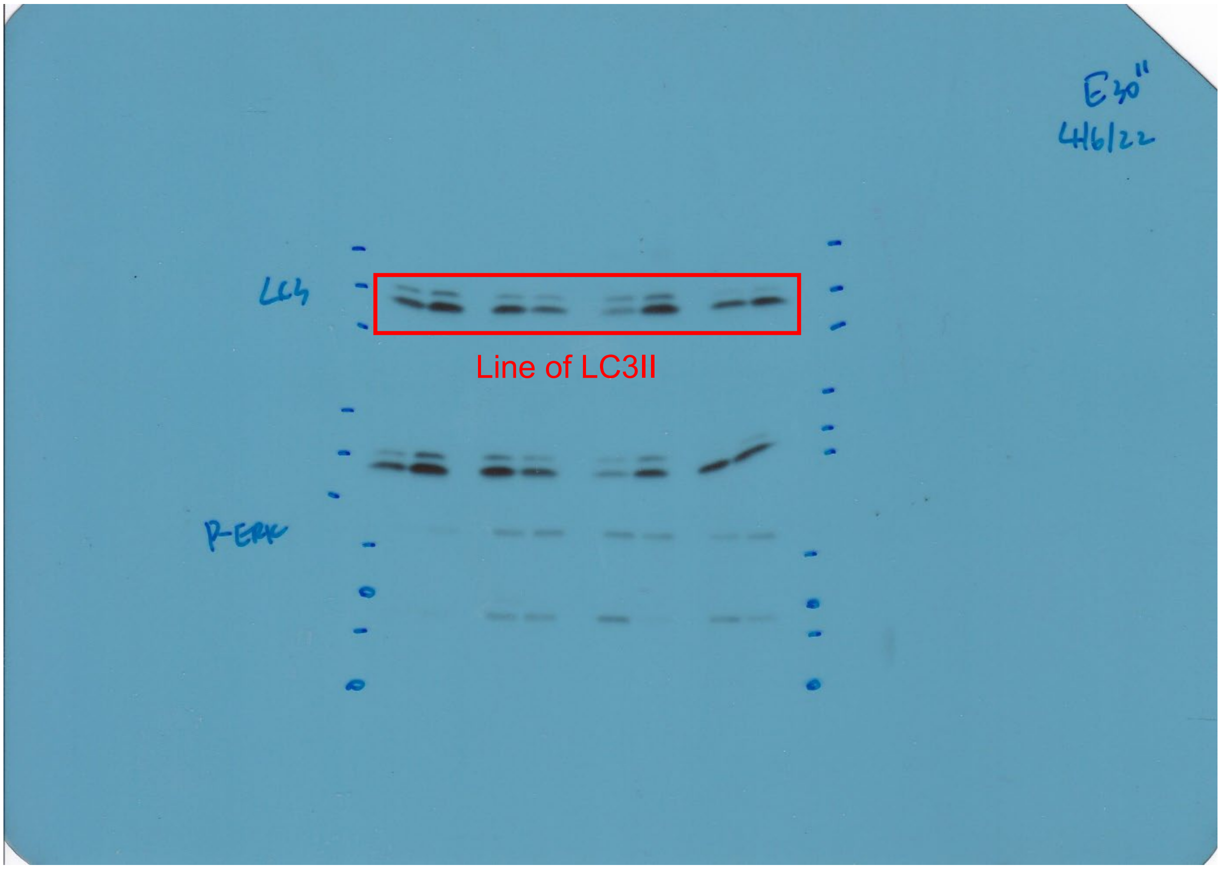

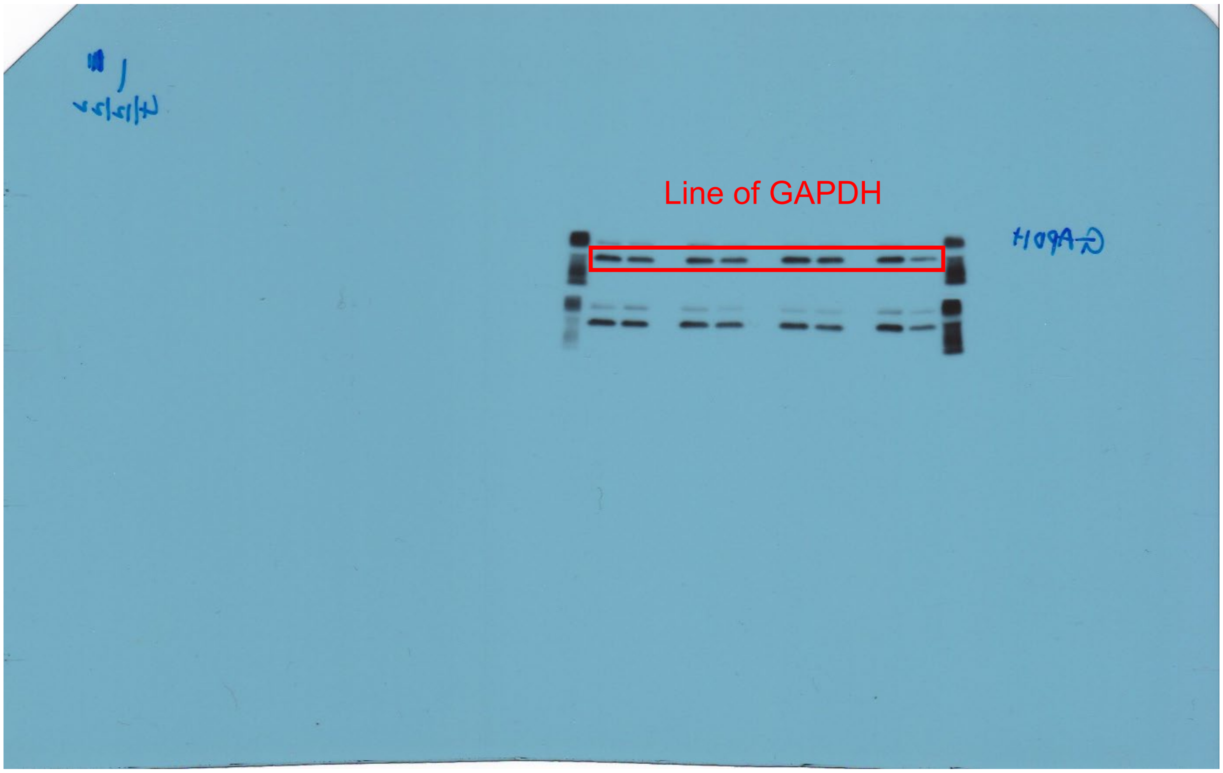

Full unedited gel for Figure S3: Drp1

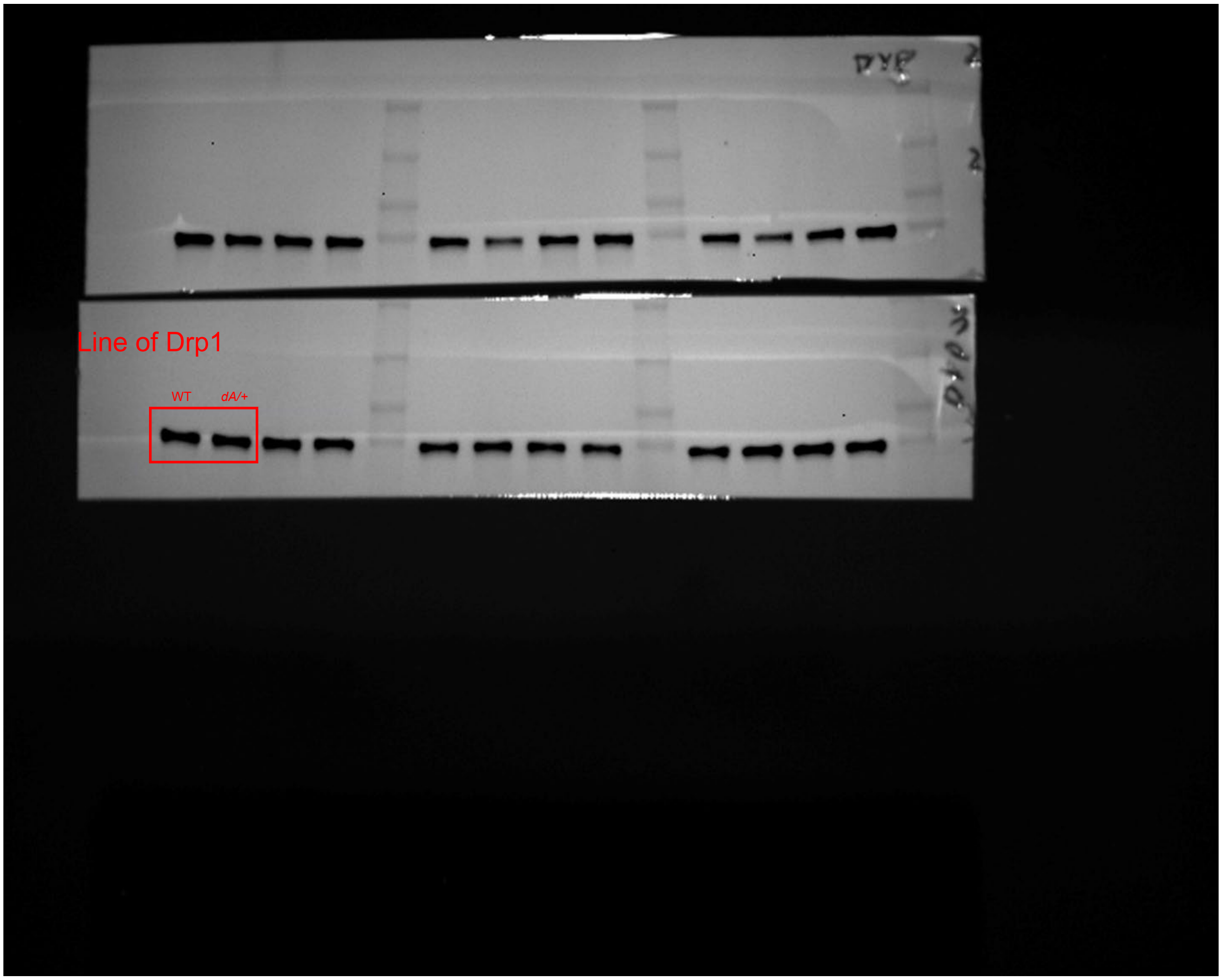

Full unedited gel for Figure S3: Tom20

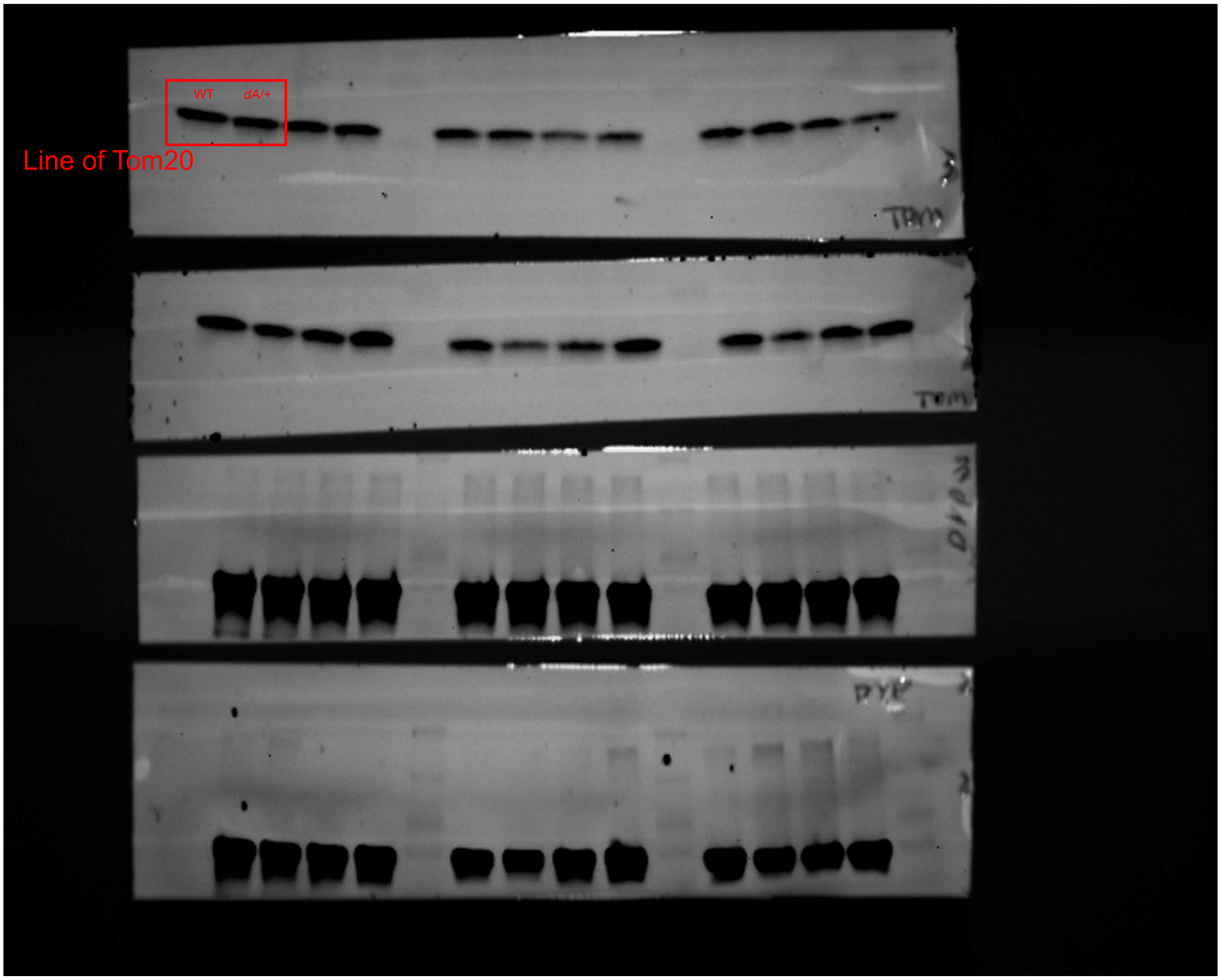

Full unedited gel for Figure S3: GAPDH

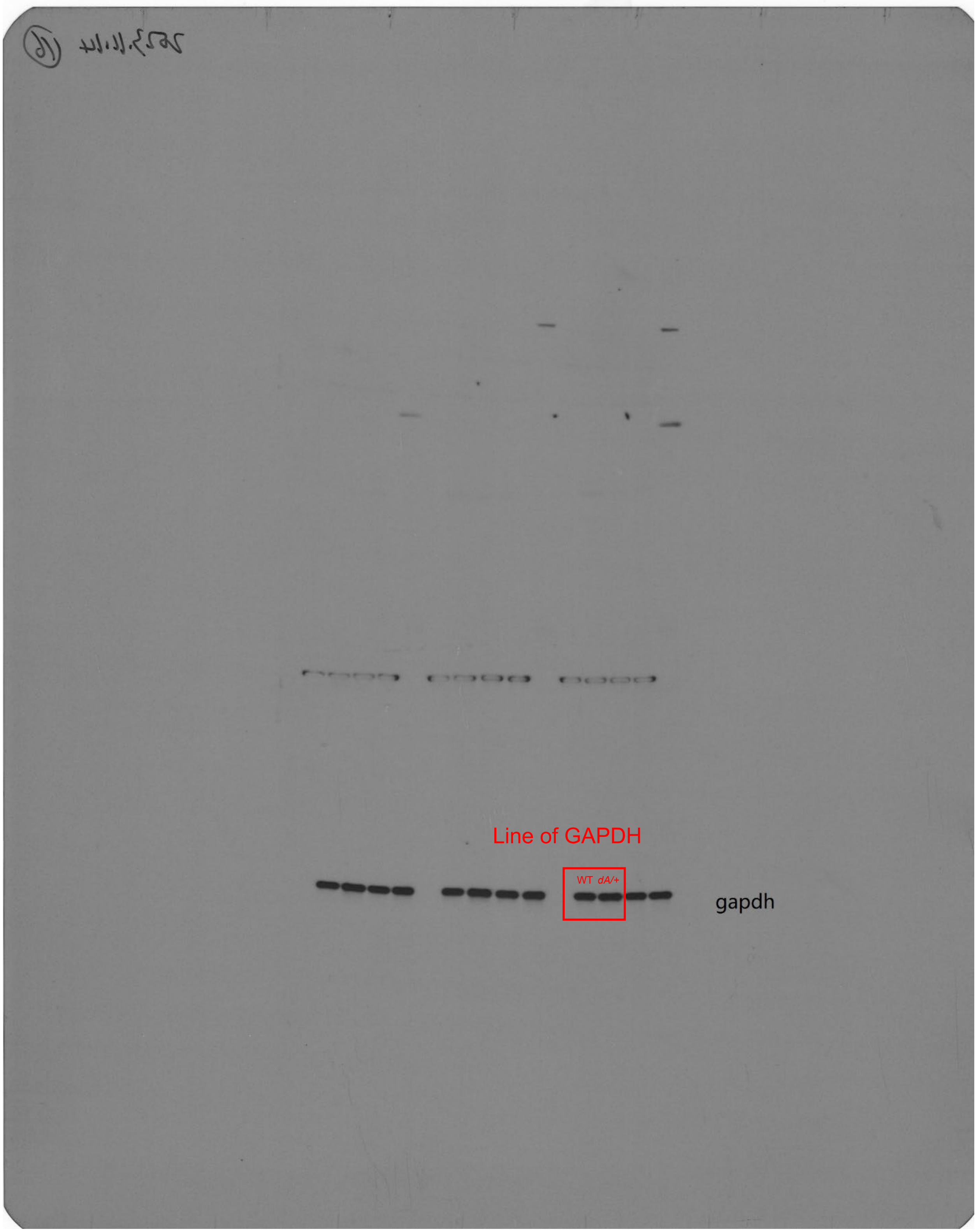

Supplement: Unedited blot and gel images [file jciinsight-9-175501-s035.pdf]
